# Comparison of prednisolone and alternative glucocorticoid dosing protocols for canine hypoadrenocorticism: insights from a survey-based study

**DOI:** 10.3389/fvets.2025.1544750

**Published:** 2025-04-14

**Authors:** Christin Emming, Anna Karoline Geks, Sajedeh Sajadihezaveh, Thomas Rieker, Johannes Brutsche, Holger Andreas Volk, Johanna Rieder

**Affiliations:** ^1^Department for Small Animal Medicine and Surgery, University of Veterinary Medicine Hannover, Hannover, Germany; ^2^AniCura Small Animal Specialists Augsburg GmbH, Augsburg, Germany; ^3^AniCura Germany GmbH, Ravensburg, Germany; ^4^Department of Mathematical Stochastics, University of Freiburg, Freiburg, Germany

**Keywords:** hypoadrenocorticism, glucocorticoid, hydrocortisone, long-term therapy, dog

## Abstract

**Objectives:**

The aim of the study was to analyze glucocorticoid (GC) dosing protocols in dogs with hypoadrenocorticism (HA), and to identify predictors for optimal clinical outcomes during both the acute and chronic phases of the disease, as well as during long-term therapy.

**Methods:**

This observational cross-sectional study utilized a case-based online questionnaire. Practicing veterinarians across Germany were invited to participate. The survey collected data in the disease course and follow-ups. Responses were analyzed using descriptive statistics, single and multiple comparisons, and a multivariable logistic regression model.

**Results:**

For 103 dogs the questionnaire was fully completed and analyzed. Of these, 85 dogs (82.5%) were hospitalized, and they received either prednisolone (52.9%), dexamethasone (31.8%) or hydrocortisone (11.8%). Hydrocortisone therapy was associated with a shorter hospitalization time and faster normalization of electrolytes compared to prednisolone. Follow-up data were available for 85 dogs, with 82.35% (*n* = 70/85) achieving an optimal or well-adjusted clinical outcome. Multivariable logistic regression analysis indicated that the eukalemic and eunatremic form was significantly less associated with the presence of azotemia and the occurrence of an acute adrenal crisis. Dividing the daily GC dosage was associated with poorer clinical outcomes and a reduced likelihood of achieving optimal medication adjustment.

**Conclusion:**

Our findings provide new, relevant recommendations for the therapeutic management of HA in dogs. Hydrocortisone appears to be a promising treatment for managing HA during hospitalization, highlighting its potential use in clinical practice. Once-daily administration of prednisolone is advisable for long-term therapy. To achieve the best possible outcome, implementing an optimal treatment protocol is essential, which veterinarians should tailor based on the needs of both owners and animals. The main limitations of the study include its retrospective nature and the limited number of participants. Future studies, particularly prospective ones, could further validate the beneficial effects of hydrocortisone and evaluate long-term therapy in comparison to prednisolone.

## 1 Introduction

Hypoadrenocorticism (HA) is an uncommon endocrinological disease in dogs, with estimated prevalences ranging from 0.3 to 1.1% (1, 2). The disorder is characterized by adrenal cortical insufficiency, leading to inefficient production of glucocorticoids (GC) and, frequently, mineralocorticoids (3, 4). Various forms of HA exist, and different subtypes and terms have been used to describe them (5). The “Agreeing Language in Veterinary Endocrinology” (ALIVE) project of the European Society for Veterinary Endocrinology (ESVE) recommends classifying HA based on electrolyte abnormalities (6).

Primary HA (adrenal insufficiency) is characterized by a complete deficiency of both GC and mineralocorticoids and is typically associated with hyperkalemia and hyponatremia (7). Secondary HA (pituitary insufficiency) refers to impaired pituitary secretion of adrenocorticotropic hormone (ACTH), resulting in reduced stimulation of the adrenal glands and primarily affecting cortisol levels. Measurement of endogenous ACTH is recommended for differentiating between primary and secondary HA (6). Approximately, 30% of dogs are presented with an acute adrenal crisis (Addisonian crisis), characterized by hypovolemic shock, bradycardia or tachycardia, collapse, hypothermia, and weak pulses at the time of diagnosis (8). This situation is life-threatening and requires hospitalization along with intensive medical care (9). Treatment of HA includes lifelong hormone replacement therapy to address the associated deficiencies. During an adrenal crisis, an increased GC dose is advised, ranging from three to 10 times the physiological requirement (8).

After overcoming the adrenal crisis and stabilizing the patient, a titration method should be used to adjust the dose to meet the individual patient's daily physiological or minimally effective replacement needs (8). During stressful situations, a temporary increase in the GC dose is advised, typically involving a 100% increase, depending on the circumstances and the patient (10). The primary goals of GC replacement therapy in patients with HA are to compensate for hormone deficiencies, prevent adrenal crises, and avoid overdosing, which may negatively impact quality of life and result in multiple comorbidities, as commonly observed in humans (11). Clinical signs of iatrogenic hypercortisolism, such as polyphagia, polydipsia, polyuria, weight gain, and changes in skin and coat condition, are frequently observed in dogs, even at low doses of GC (12, 13). A retrospective study of Kintzer et al. found that 31% of dogs with primary HA exhibited adverse effects associated with iatrogenic HA during long-term treatment (14). Optimizing treatment for affected dogs can therefore be challenging. However, the same study reported that 86.5% of the dogs had a good to excellent response to treatment. Additionally, the study indicated a median survival time of 4.7 years, suggesting a good prognosis (14).

To the best of the author's knowledge, no laboratory parameters exist that reliably monitor under- or overtreatment with GC (11, 15). One retrospective study evaluating endogenous ACTH levels in dogs with HA during treatment suggested that concentrations below the detection threshold are likely to exclude GC deficiency. Although correct preanalytical procedures are crucial due to the fragile nature of canine endogenous ACTH (12). Nonetheless, the available information on GC treatment regimens for both acute and long-term therapy is limited and outdated. The relationship between the GC dose and factors such as signalment, type of adrenal insufficiency, hospitalization period, quality of life, and life expectancy has not been studied in detail. In recent years, research has primarily focused on the management of mineralocorticoid replacement therapy. After the approval of DOCP (Zycortal^®^, Dechra) in the European Union in 2015, numerous studies were conducted to determine its optimal dosage (16–19). Given that GC substitution is essential for all forms of HA, the limited number of studies on GC therapy is particularly concerning.

The objective of this study was to evaluate GC replacement therapy in dogs with HA, distinguishing between treatment during an acute adrenal crisis and long-term disease management. To achieve this, a comprehensive survey was designed for veterinarians, encompassing a large number of cases to provide valuable insights into GC dosing strategies. Firstly, we aimed to analyze GC dosing regimens during hospitalization at the onset of the disease and to investigate how appropriate therapy affects the initial clinical outcome and recovery. Secondly, we sought to identify predictors associated with optimal overall condition during long-term therapy. Using the collected data, various GC protocols during acute adrenal crisis hospitalization were compared. Based on these findings, specific and useful recommendations were formulated to optimally support veterinarians in their treatment decision-making. Initially, the authors hypothesized that hydrocortisone, the first-line agent in human medicine, could be a beneficial treatment option for dogs, a hypothesis that is further supported by the present study. Additionally, we suspected that many dogs are prescribed continuously high doses of GC, and their treatment may be suboptimally adjusted. Our results highlight that HA is associated with a generally favorable prognosis, with the largest proportion of dogs being well to optimally adjusted. One predictor could be identified, which was statistically associated with an increased likelihood of achieving optimal treatment adjustment for long-term therapy.

## 2 Methods

### 2.1 Study design

An observational cross-sectional study was designed to retrospectively gain information about treatment protocols and clinical outcomes from dogs diagnosed during a defined period (2016 to 2023). To achieve our goals, an online case-based questionnaire for veterinarians was created and sent out to small animal veterinary clinical professionals across Germany. The software platform Lime Survey Version 2.05+ (Lime Survey, Hamburg, Germany), an electronic data capture system for clinical trials, was used. Participation was anonymous and voluntary. The data collection for this survey was subject to the General Data Protection Regulation (GDPR). Participants had to accept our privacy policy before starting the survey. The case-based data were stored on the server of the University of Veterinary Medicine Hannover and were accessible only to the main author. Veterinarians were invited to participate through a combination of advertisements on the social media pages of the University of Veterinary Medicine Hannover (Facebook, Instagram) and personal contact with the main author. The focus was on large referral centers with board-certified specialists to generate a high number of cases and ensure high-quality treatment, which probably resulted in selection bias. Nevertheless, all practicing veterinarians from various sectors (veterinary practices, referral centers, university clinics) were invited to participate in this study to prevent selection bias and ensure sufficient generalizability. The questionnaire was online and accessible from February 2023 to March 2024.

### 2.2 Questionnaire

The survey was conducted exclusively by veterinarians and was based on their self-reported statements and records. The questionnaire encompassed multiple-choice questions, filtered questions, and open-ended questions. The first part addressed general questions about signalement, survival, and death. The age (in years) of each case was calculated based on the date of birth and the date of initial diagnosis. The second section focused on the initial clinical signs, laboratory abnormalities, diagnosis of HA, and HA type. The third part included information about in-house treatment. The questions related to GC therapy included the type of GC used, the exact dosage, the form of application, the frequency of administration, and possible adjustments during hospitalization. Additionally, questions were asked about the duration of hospitalization, the time required for electrolyte normalization, and the time needed for stabilization of the general condition. The response options for the duration of hospitalization were 24, 48, 72, 96, 120, and more than 120 h. For the normalization of electrolytes, the available response options were 6, 12, 24, 48, 72 h, and more than 72 h. For the stabilization of the general condition, the response options included 12, 24, 48, 72, and more than 72 h. The fourth part was available for veterinarians who continued the follow-up of the patient and pertained to long-term therapy (GC and/or mineralocorticoids) and potential adjustments. If the case involved the disease lasting longer than 2 years, answers to questions were limited to the first 2 years and did not reflect the entire course of the disease. The last part included questions about additional comorbidities associated with HA. All corresponding drug doses had to be entered manually in mg/kg/d. Furthermore, the veterinarian was asked about clinical signs of over- or underdosing and the quality of life. The questionnaire is available in the Supplementary material (in German and English).

### 2.3 Inclusion criteria

Data were included if the HA diagnosis was made between 2016 and 2023 and was based on an adequate test procedure (e.g., ACTH stimulation test). The dog should have been receiving treatment for a minimum of 3 months. According to this, all dogs were excluded if the diagnosis was made before 2016, they had been under therapy for less than 3 months, no corresponding test evidence was available, and the responses were incomplete or ineligible. Specific GC doses were excluded if a coexisting disease influenced the actual, individualized dose. The year 2016 was specifically chosen because DOCP (Zycortal^®^, Dechra) was approved in the European Union in November 2015. The authors assume that, as a result, many dogs were additionally treated with this medication, leading to reduced variability and increased homogeneity. Furthermore, this treatment method, involving monthly injections and regular electrolyte monitoring, is associated with a higher number of follow-ups and greater documentation obligation, which may help minimize recall bias.

### 2.4 Statistical analysis

Collected data were recorded in Microsoft Excel files. GraphPad Prism version 10 (GraphPad Software Inc., CA, USA) was used for descriptive statistical analyses and comparison tests. Shapiro-Wilk tests were used to assess the normality of the data. The Wald method was used to calculate prevalences, and the resulting data were presented as frequencies (n), percentages, and 95% confidence intervals (CI). In the descriptive statistics, mean ± standard deviation (SD) was used to describe normally distributed data, while median ± interquartile range (IQR) was used to describe non-normally distributed data. IQR represents the range between the 25th and 75th percentiles of the data. The Wilcoxon signed-rank test was used to contrast the body weight at the time of HA diagnosis and at the follow-up. The Friedman test and Dunn's multiple comparisons test were applied to evaluate the prednisolone doses of specific patients at specific time points during the hospitalization period. The comparison of two groups was performed using the Mann-Whitney test for non-parametric data and the Unpaired *t*-test for parametric data. Results from several groups were compared using the Kruskal-Wallis test (non-parametric data, in conjunction with Dunn's multiple comparisons test) or the Ordinary One-way ANOVA (parametric data, in conjunction with Tukey's multiple comparisons test).

In particular, the hospitalized patient groups treated with either a prednisolone derivative, dexamethasone, or hydrocortisone were compared in terms of hospitalization time, speed of electrolyte normalization, and normalization of general condition. As mentioned above, the questionnaire contained specific time points and used a critical value that exceeded the previous time interval. Since no explicit time was provided, the next higher time period was used for statistical analysis in cases where participants selected the highest time interval (e.g., “more than 120 h” was treated as 144 h).

Multinomial logistic regression analysis, implemented using Python's statsmodels.api MNLogit function, was employed to identify key clinical predictors of treatment outcomes, such as hospitalization duration and electrolyte normalization speed. By the design of the questionnaire, these variables were reported as different classes which were grouped as short/long and fast/slow, respectively, resulting in a classification problem with two possible outcomes. Furthermore, the analysis aimed to explore relationships between clinical outcomes during long-term therapy and various independent variables (variant of HA group, current or last GC, current or last GC dosage, current or last GC divided into). The optimal, well, or moderate medication adjustment of the patient, with a particular focus on GC treatment, was considered as the dependent variable. The following parameters are specified for the regression analysis: regression coefficient (coef), 95% CI, standard error (std err), odds ratio (OR), and *p*-value. A final multivariable model was created considering major dependent variables, and its discriminatory power was assessed using the area under the receiver operating characteristic curve (AUROC). The AUROC values were interpreted as follows: more than 90% indicates a highly accurate test; between 80 and 90% a moderately accurate test; between 70 and 80% a fairly accurate test; and below 70% a poorly accurate test (20). Statistical significance was set at *p* < 0.05. The receiver operating characteristic curve (AUROC) was used to assess the validity of the following dependent variables: short hospitalization period, rapid normalization of electrolytes, and specific HA variant.

## 3 Results

### 3.1 Patient characteristics

Two hundred sixty-seven cases were available for analysis, of which 103 questionnaires were complete. As an inclusion criterion, an ACTH stimulation test was performed on all dogs enrolled in the study. Additional basal ACTH measurements were conducted in four patients (3.9%), and aldosterone testing was performed in nine dogs (8.7%). Fifty-four dogs were male (32 neutered), and 49 were female (35 spayed). The median age at the time of diagnosis was 4.9 years (IQR: 3.2–13.3 years), and the median body weight of the dogs was 17.75 kg (IQR: 8.70–22.05 kg). The dataset comprised 39 crossbreeds (37.9%) and 64 purebred dogs (62.1%). The most frequent breeds were the Jack Russell Terrier, Labrador Retriever, and Australian Shepherd (*n* = 4 for each). These were followed by the Poodle, Chihuahua, and West Highland White Terrier (*n* = 3 for each). At the time of initial presentation, electrolyte disturbances (hyperkalemia and/or hyponatremia) were observed in 86.41% of the study population, indicating GC and mineralocorticoid insufficiency (*n* = 89; 95% CI: 78.35–91.85). No electrolyte abnormalities were found in 13.6% (*n* = 14; 95% CI: 8.2–21.6%).

### 3.2 Management of dogs at the time of HA diagnosis

Within this cohort, outpatient therapy was provided to 18 out of 103 dogs (17.5%), including two on day-care treatment. Eighty-five dogs (82.5%) were hospitalized for at least 24 h, and among these, the majority (78.8%; *n* = 67) had an acute adrenal crisis. The hospitalized dogs were subdivided according to their initial GC therapy into three categories: prednisolone derivative, dexamethasone, and hydrocortisone. Prednisolone was the treatment of choice in 52.9% (*n* = 45) with a median starting dose of 0.5 mg/kg/d (day) (IQR: 0.36–1.08 mg/kg/d). The following routes of administration were used: 60% intravenous (IV; *n* = 27), 35.6% oral (PO), and 2.2% intramuscular (*n* = 1). The majority of dogs received the dose once daily (*n* = 36, 80%), while 17.8% were administered the dose in two divided doses. For one patient, the route and frequency of administration were not specified. In total, dexamethasone was administered to 27 dogs; however, one dog was excluded due to concurrent immune-mediated hemolytic anemia. Consequently, dexamethasone was given to 30.6% of the dogs (*n* = 26), with an initial median dose of 0.2 mg/kg/d (IQR: 0.1–0.2 mg/kg/d). Of these, 84.6% (*n* = 22) received the drug IV, while the remaining 15.4% (*n* = 4) were treated subcutaneously (SC). A once-daily administration was recorded in 92.2% of cases (*n* = 24), while one patient received the medication twice daily and one patient three times daily. Hydrocortisone therapy was given to 11.76% of the dogs (*n* = 10), either as a continuous drip (mean 0.5 mg/kg/h) or as bolus therapy (mean 2.62 mg/kg/d; SD 1.51).

The median hospitalization time was 72 h in the prednisolone derivative group (IQR: 48–120 h), and 48 h in both the dexamethasone group (IQR: 48–72 h) and the hydrocortisone group (IQR: 24–72 h). The median duration for electrolyte normalization was 48 h across all groups, with an IQR of 24–96 h in the prednisolone derivative group, 24–72 h in the dexamethasone group, and 12–48 h in the hydrocortisone group.

**Figure 1:**
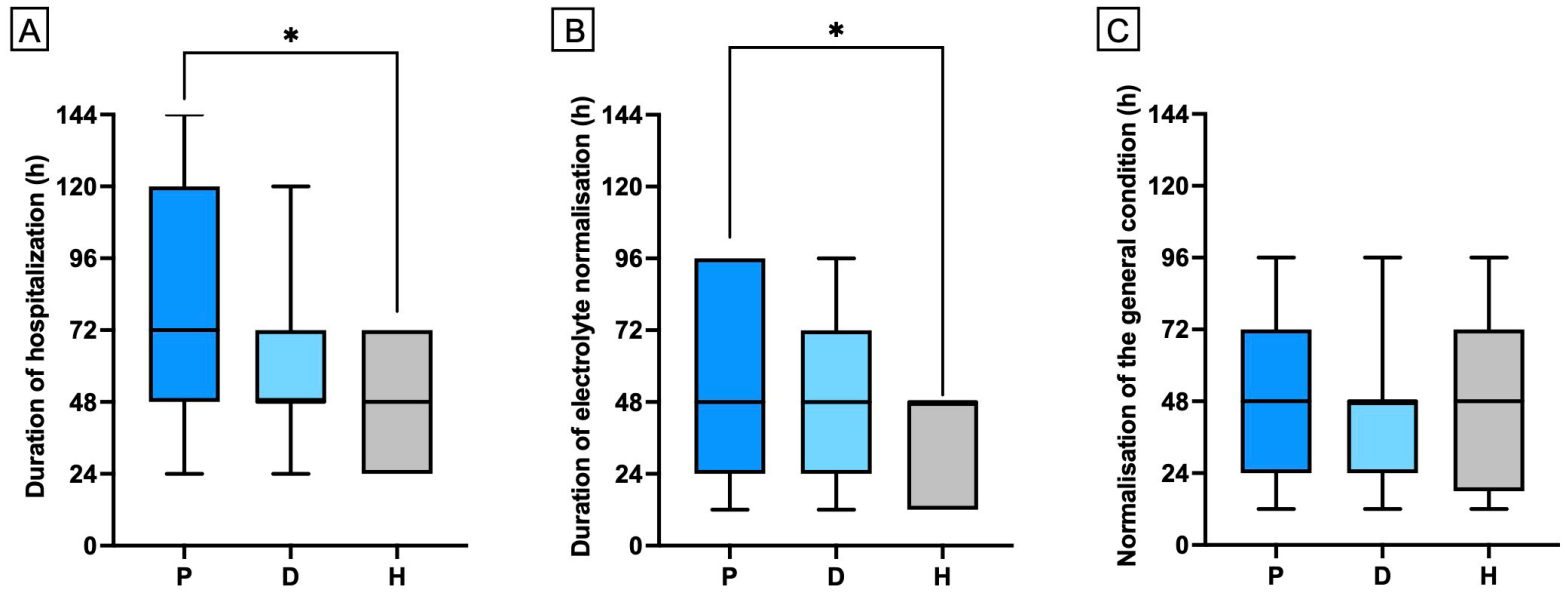
Comparison of hospitalization period **(A)**, time to normalization of electrolytes **(B)** and time to normalization of general condition **(C)** among dogs receiving either prednisolone derivative (P; *n* = 5), dexamethasone (D; *n* = 26), or hydrocortisone (H; *n* = 10). Data are presented as bar graphs showing the mean and standard deviation (Mean ± SD). Asterisks (*) indicate statistically significant differences between groups (*p* ≤ 0.05).

**Figure 2:**
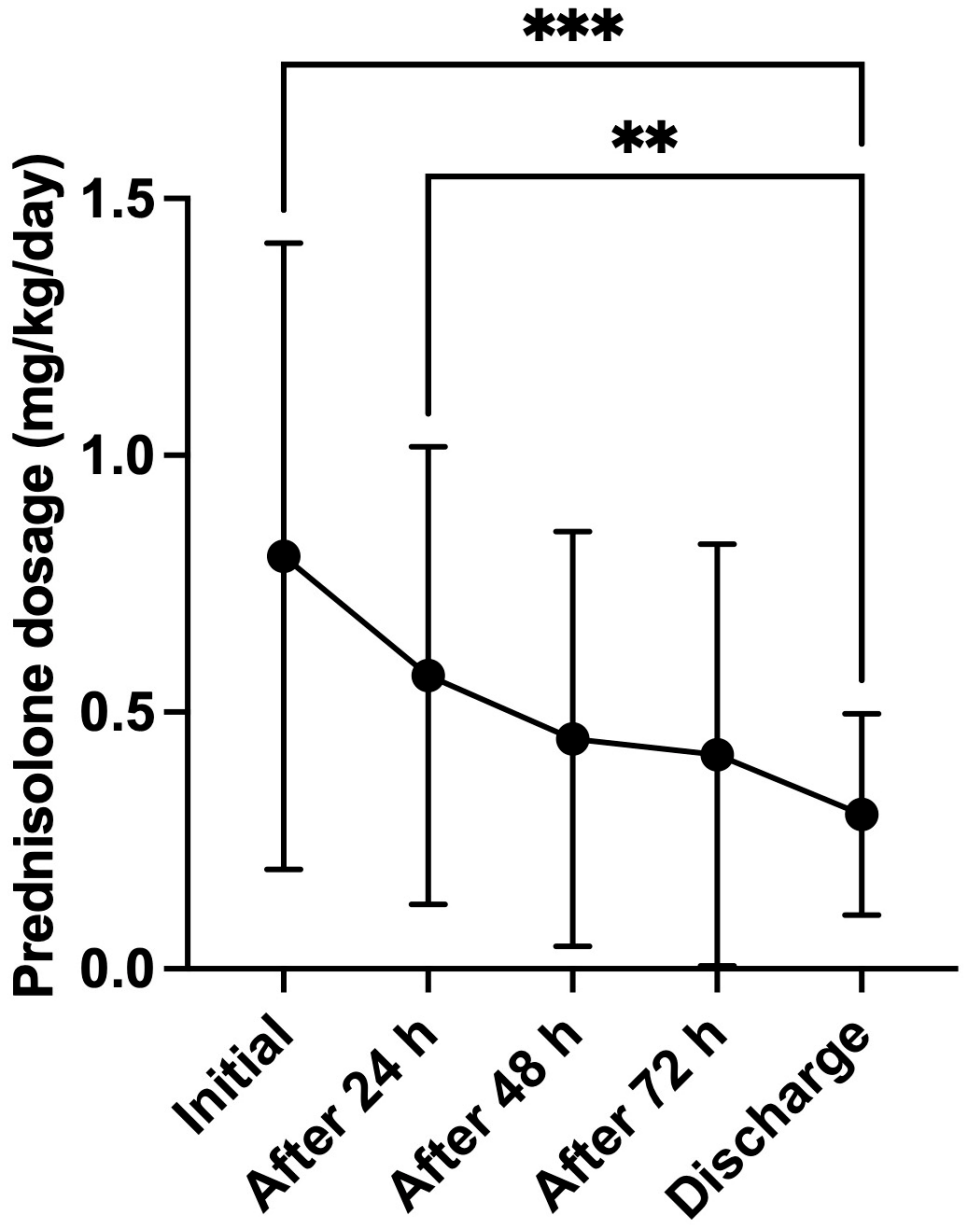
Development of the prednisolone dosage (mg/kg body weight/day) over the complete hospitalization period (minimum 72 h) in dogs (*n* = 19), treated with a prednisolone derivative. Data are presented as mean ± SD with error bars. Asterisks (*) indicate statistical significance: **p ≤ 0.01, ***p ≤ 0.001.

Hydrocortisone treatment was associated with significantly shorter hospitalization times and faster electrolyte normalization compared to prednisolone (Figure 1; *p* = 0.012, and *p* = 0.046, respectively). No significant differences were observed between the treatment groups regarding the improvement of the clinical condition (Figure 1; *p* = 0.9496), or in age and weight at the time of HA diagnosis (*p* = 0.1000, and *p* = 0.4024, respectively). At discharge, the median prednisolone dose was 0.28 mg/kg/d in 84 dogs (IQR: 0.2–0.5 mg/kg/d). Nineteen hospitalized dogs treated with a prednisolone derivative had their dose significantly reduced during their stay of at least 72 h, reaching the discharge dose (Figure 2, *p* = < 0.0001).

### 3.3 Monitoring during HA treatment

Follow-up data was available for 85 out of 103 dogs. An adjustment of the GC treatment protocol was performed based on individual patient needs in 91.8% of the study population (*n* = 78). All dogs who were receiving continued cortisol replacement therapy were first given prednisolone. A reduction in the prednisolone dose was the most frequent adjustment, occurring in 94.9% of the dogs (*n* = 74). This included single reductions in 18.9% of the cases (*n* = 14) and repeated adjustments in 78.4% of the patients (*n* = 58). Conversely, a dose increase was made in 20.5% of the dogs (*n* = 16). A change in medication was conducted in 10.26% of the cases (*n* = 8), with four dogs being switched from prednisolone to hydrocortisone. At the time of the study, the current prednisolone dose was 0.11 mg/kg/d for 79 dogs (IQR: 0.07–0.18 mg/kg/d).

**Figure 3:**
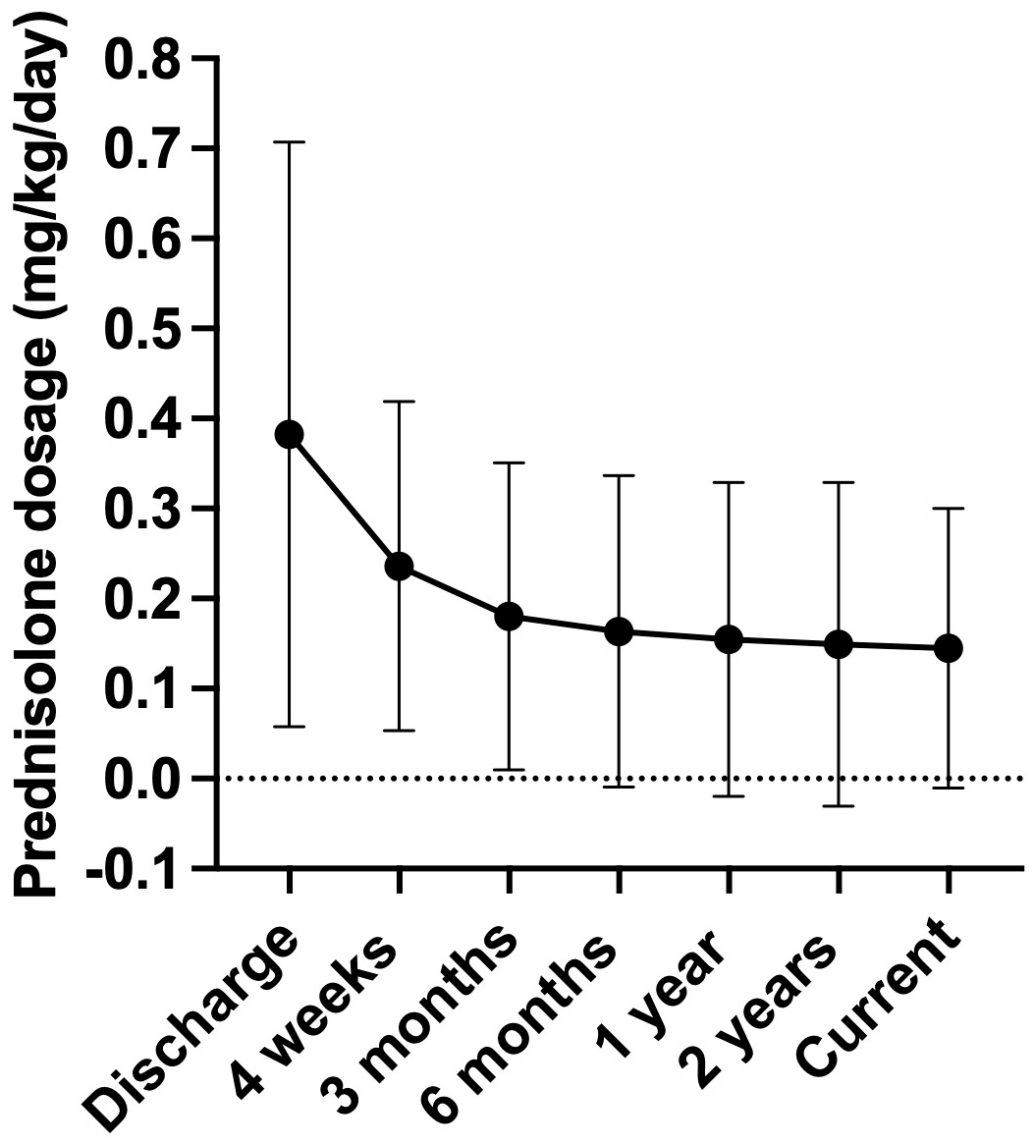
Development of the prednisolone dosage (mg/kg body weight/day) during long-term therapy. The X-axis represents the different time points: discharge after hospitalization (*n* = 83), 4 weeks post diagnosis (*n* = 18), 3 months post diagnosis (*n* = 75), 6 months post diagnosis (*n* = 71), 1 year post diagnosis (*n* = 66), 2 years post diagnosis (*n* = 62), and the current dose at the time of questionnaire completion (*n* = 79). Data were not available for all dogs up to 2 years post diagnosis, as some dogs were not under therapy for that long or were treated by another veterinarian and lost to follow-up. Data are presented as mean ± SD with error bars.

The prednisolone doses at discharge, as well as at specific time points after discharge (4 weeks, 3 months, 6 months, 1 year, 2 years), and at the time of questionnaire completion were compared both overall and at each respective time point. Overall, a significant reduction in the prednisolone dose was observed over the disease period (Figure 3, *p* = < 0.0001). The prednisolone dose administered after 4 weeks differed significantly from the doses administered after 6 months, 1 year, 2 years, and at the time of questionnaire completion (*p* = 0.004, *p* = 0.0006, *p* = < 0.0001, and *p* = 0.0002, respectively). From 3 months post–HA diagnosis, no further significant dose reduction was observed.

Of the dogs, 91.1% (*n* = 72) were treated with both a GC and a mineralocorticoid. Among them, 13 dogs received fludrocortisone, and 59 dogs were treated with DOCP injections. Conversely, 8.9% of the dogs (*n* = 7) received no additional mineralocorticoid replacement therapy. Most of these dogs initially presented with physiological potassium and sodium concentrations, except for one. There was no significant difference in the permanent prednisolone dose between dogs receiving or not receiving mineralocorticoid medication (*p* = 0.416). The prednisolone dose at the point of questionnaire completion was compared between groups defined by the initial electrolyte imbalances. No significant difference was found based on the type of HA (*p* = 0.962) or the type of GC used during hospitalization (*p* = 0.336).

### 3.4 Outcome

Fifty-six dogs (54.37%) were still alive at the end of the study. Eight dogs (7.8%) died during the course of the disease, with only one dog (1.0%) dying due to an adrenal crisis. The outcome was unknown for 39 dogs (37.86%). A significant weight increase was reported in many dogs (*p* = < 0.0001). Quality of life and side-effect profiles were available for 82 dogs. Optimal adjustment was reported for 39 dogs (47.6%). Thirty-one dogs (37.8%) were well-adjusted, and 12 dogs (14.6%) were moderately adjusted.

### 3.5 Comorbidities

Coexisting diseases were noted in 39.0% of the dogs, with at least one additional condition was observed in 30 dogs. These included immune-mediated disorders (*n* = 8), endocrinological diseases (*n* = 5), gastrointestinal diseases (*n* = 8), neurological disorders (*n* = 4), dermatological disorders (*n* = 4), and neoplastic diseases (*n* = 5). In five dogs, the dose of prednisolone was increased beyond the physiological level due to the presence of an additional disease. Among these five dogs, four had immune-mediated disorders, and one had intracranial neoplasia.

### 3.6 Multinomial logistic regression analysis

The data from the multinomial logistic regression analysis are presented in Table 1. The presence of an acute adrenal crisis and azotemia are significantly associated with a lower likelihood of the eukalemic/eunatremic form of HA (*p* = 0.008; OR: 0.167; 95% CI: 0.044–0.630, and *p* = 0.093; OR: 0.093; 95% CI: 0.019–0.464), respectively). The calculated OR further indicates that acute adrenal crises reduce the OR of an eukalemic/eunatremic presentation by 83.3%, while azotemia reduces it by 90.7%. This model presents a strong discriminatory power (AUROC = 0.743). The once-daily administration of prednisolone is significantly associated with a higher probability of achieving an optimal medication adjustment (*p* = 0.019; OR: 6.63; 95% CI: 1.36–32.34). In contrast, dividing the daily GC dosage negatively impacts clinical outcomes and reduces the likelihood of an optimal medication adjustment. The OR of 6.63 indicates a 563% higher probability of having an optimal outcome in GC therapy when prednisolone is administered once daily. The AUROC score indicates a low discriminatory effect (AUROC = 0.610).

**Table 1:** Multivariable logistic regression model assessing associations between specific HA forms and clinical outcomes in dogs with HA throughout the disease course.

**Variable: dependent**	**Variable: independent**	**Coefficient**	**Standard Error**	**Statistical comparison (*p*-value)**	**Odds ratio (OR)**	**95% confidence interval (CI)**	**AUROC score**
Eukalemic/ eunatremic form	Acute adrenal ciris	−1.7906	0.677	0.008	0.167	0.044–0.630	0.743
	Azotemia	−2.3712	0.818	0.004	0.093	0.019–0.464	
Optimal outcome (Grade 1)	Once daily administration of prednisolone	1.8928	0.809	0.019	6.64	1.36–32.34	0.610

The following dependent variables were analyzed and eliminated during multivariable logistic regression: hyperkalemic and/or hyponatremic form, eukalemic/eunatremic HA form, optimal/ good/ moderate clinical outcome regarding the last/current GC dose. The receiver operating characteristic curve (AUROC) was used to assess the validity. Statistical significance was set at p ≤ 0.05.

## 4 Discussion

This retrospective study, involving data from 103 dogs, provides new insights into GC therapy for the initial and long-term management of HA. Our findings suggest that hydrocortisone is a valuable option during initial hospitalization and may offer advantages over prednisolone in certain treatment aspects. The multinomial logistic regression analysis identified that the once-daily administration of prednisolone increases the likelihood of an optimal clinical outcome. This study extends current knowledge and offers deeper insights into optimizing GC dosing for dogs with adrenal insufficiency.

Epidemiologic data were consistent with existing literature concerning breed prevalence, age, and sex (15, 21, 22). The ACTH stimulation test, the gold standard for diagnosing HA, was performed on all enrolled dogs (23). The majority of cases (86.41%) presented with hyperkalemic and/or hyponatremic HA, indicative of primary HA associated with combined GC- and mineralocorticoid deficiency. Aldosterone deficiency causes decreased potassium excretion and retention of hydrogen ions, leading to hyperkalemia and metabolic acidosis. Additionally, sodium and water depletion through the kidneys results in hyponatremia and dehydration (3, 13, 24). Increased plasma concentrations of arginine vasopressin (AVP) can also impair water excretion. Despite the presence of hypoosmolality in HA patients, the occurrence of hypovolemia provides a strong non-osmotic stimulus, and the central negative feedback mechanism of GC on AVP release is absent, leading to further sodium dilution due to water retention and dilution (10, 25, 26). In 13.59% of the dogs, eukalemic/eunatremic HA was observed, which is consistent with previous reports indicating that 1 to 30% of dogs with primary HA have electrolyte concentrations within the reference range at the time of diagnosis (27–29). Less commonly, secondary HA involving the pituitary gland or GC-isolated HA resulting from selective destruction of the zona fasciculata and zona reticularis has been described in some canine reports (30). However, due to the lack of endogenous ACTH and aldosterone measurements, further categorization of these animals was not feasible, and they were classified as either hyperkalemic and/or hyponatremic or eukalemic/eunatremic.

Out of 103 dogs, 85 were monitored as inpatients. Most of these dogs (78.8%) experienced an acute adrenal crisis, a higher rate compared to previous reports (31). This discrepancy may be attributed to improved diagnostic capabilities and increased sensitivity of emergency clinicians to the disease. The primary treatment for an adrenal crisis involves two key components: IV fluid resuscitation, and cortisol replacement therapy (9). Balanced isotonic electrolyte solutions are recommended for correcting hypovolemia, electrolyte imbalances, and acid-base disturbances, as they act more quickly than 0.9% NaCl in terms of acid-base normalization and reduce the risk of overly rapid sodium correction in hyponatremic patients (32). In humans, daily adrenal cortisol production of 5–6 mg/m^2^ serves as a guideline for maintenance cortisol replacement in GC deficiency cases (33, 34). Such specific information is lacking in veterinary medicine, particularly concerning body surface area, which is infrequently used for GC dosing. However, recent research suggests that dogs with higher body weights may be at an increased risk of experiencing side effects from GCs (35). Cortisol replacement therapy involves various protocols differing in administration route, potency, duration of action, and residual mineralocorticoid activity compared to endogenous cortisol (36). Hydrocortisone, a short-acting analog of endogenous cortisol, has a biological half-life of approximately 8–12 h. Prednisolone, an intermediate-acting GC, has a half-life of 12–36 h and is roughly 4 to 5 times more potent than hydrocortisone (37). In the literature, the estimated physiological dose of prednisolone for dogs is reported as 0.05 to 0.2 mg/kg/d (12, 13). Long-acting GCs like dexamethasone are 4 to 10 times more potent than prednisolone and have a duration of action exceeding 48 h (37).

Our study found that prednisolone was the most frequently used hormone replacement therapy in Germany, accounting for 52.9% of cases during an adrenal crisis. In contrast, dexamethasone was the most common GC treatment for adrenal crises in the United Kingdom (18). In human medicine, prednisolone is used as a second-line option, with dexamethasone being less preferred (38). Long-acting GC agents are associated with a higher risk of side effects in patients with HA (36). A recent study in humans found that long-acting synthetic steroids (e.g., prednisolone, dexamethasone), used in people with hypopituitarism, are linked to a higher risk of adverse metabolic effects such as weight gain, dyslipidemia, and diabetes mellitus (39). The median hospitalization duration in our study was 2 to 3 days, depending on the respective GC administration. These data are similar to other recent studies, where dogs were also hospitalized for an average of 2 to 4 days (10, 40, 41). In an older study involving 38 dogs with HA, the hospitalization duration ranged from 2 to 5 days (31).

Our data showed that hydrocortisone treatment led to a better response rate among hospitalized dogs, allowing them to be discharged 1 day earlier compared to those in the prednisolone derivative group (2 days vs. 3 days). While some other retrospective studies have reported a good response rate to hydrocortisone during an acute adrenal crisis in dogs, its efficacy has not been shown to be superior to prednisolone or dexamethasone (10, 40, 41). One study found that hydrocortisone had a non-significant but slightly shorter hospitalization time (median 48 h) compared to dexamethasone (median 57 h) (40). Additionally, dogs treated with hydrocortisone in our study had a statistically significantly faster normalization of electrolytes compared to the prednisolone group. However, there was no statistically significant difference between the dexamethasone and hydrocortisone groups regarding the length of hospitalization and the time required for electrolyte normalization. The range in the hydrocortisone group was more consistent for both parameters. The study by Mitropoulou et al. found that not all dogs treated with prednisolone or dexamethasone achieved eunatremia during hospitalization, whereas all dogs receiving hydrocortisone, except for one dog with slight hyponatremia, achieved eunatremia prior to discharge. This indicates a faster normalization, although the difference was not statistically significant (41). The study by Brunori et al. did not identify a significant difference in the rate of electrolyte normalization between dogs treated with hydrocortisone and those treated with dexamethasone (40). This indicates that our study supports the effectiveness of hydrocortisone and was able to demonstrate its superiority compared to prednisolone. In humans, hydrocortisone is the first-line treatment for adrenal crises and long-term management (38, 42, 43). It has equal mineralocorticoid and GC activities. Prednisolone has about one-fourth the mineralocorticoid activity of hydrocortisone but is still ten times more potent than dexamethasone (44). In contrast, fludrocortisone is 200 to 400 times more potent as a mineralocorticoid than hydrocortisone.

Osmotic demyelination syndrome is a potentially life-threatening complication of fluid resuscitation in dogs, arising from the rapid movement of water out of brain cells due to the rapid correction of chronic hyponatremia, causing shrinkage and apoptosis of these cells, accompanied with transient or permanent neurological signs (45). The correction of hyponatremia can be achieved through the key components mentioned above. IV fluid therapy provides electrolytes and, through rehydration, abolishes the non-osmotic stimulus for AVP (10). Additionally, GC therapy initiates a negative feedback mechanism on AVP release (26). In the study by Gunn et al., 30 dogs treated with hydrocortisone, in which one dog developed osmotic demyelination syndrome due to a rapid increase in sodium levels (10). However, additional isolated reports exist in the literature describing dogs that suffered from this complication after receiving other GC preparations (e.g., prednisolone, dexamethasone) (45, 46) or even in the context of underlying conditions (e.g., Trichuris vulpis infection) (47). Therefore, it can be stated that the occurrence of osmotic demyelination syndrome represents a general complication of HA treatment in dogs, with a variety of factors contributing to its development. Nevertheless, it is advisable to delay the administration of mineralocorticoids until the animal's general condition and electrolyte levels have stabilized, due to the high potency fludrocortisone (44). In this context, another advantage of hydrocortisone is its method of administration, which, considering its short half-life, allows for flexibility. Hydrocortisone can be administered via continuous infusion or multiple doses, enabling precise dose adjustments based on vital signs and laboratory parameters, while minimizing adverse effects from improper administration (38, 40). All these characteristics suggest that hydrocortisone is a beneficial therapeutic option in dogs. The faster clinical rehabilitation of animals from the acute adrenal crisis not only provides medical benefits but also improves cost efficiency and reduces caregiver burden. A shorter hospitalization period lowers overall treatment costs, making therapy more accessible for owners. Caregiver burden can be alleviated by factors such as the remission or stabilization of a chronic disease or the ability to finance treatment costs (48). A questionnaire-based study with owners of dogs affected by HA revealed that approximately two-thirds of respondents were worried about high veterinary costs (21). The financial burden is significant and often associated with ethical and moral conflicts. A rapid recovery and earlier discharge into home care can help mitigate these financial and emotional strains, ultimately improving both the owner's well-being and the long-term treatment adherence for the animal. However, local regulatory requirements must also be considered, as there are no veterinary-licensed hydrocortisone products.

For long-term therapy, all enrolled dogs initially received prednisolone as part of their maintenance treatment. This aligns with current practice, indicating that prednisolone remains the most commonly used replacement therapy in Germany (21, 29). We observed that dose reduction was most common, with a steady dose achieved after 3 months post-diagnosis. Medication transitions were infrequent, occurring in 10.3% of cases, with hydrocortisone being used in four dogs. A recent study found that 6% of dogs were switched to hydrocortisone, primarily from prednisolone, and noted a reduction in side effects (21). In our study, no significant differences in prednisolone dosing related to the type of mineralocorticoid replacement therapy were observed. However, three dogs receiving only fludrocortisone did not require additional GC supplementation, which aligns with literature suggesting that fludrocortisone's GC potency may suffice in some cases (15). A comparison of different HA groups, based on initial electrolyte imbalances, revealed no differences in dosages, suggesting that GC dosing is independent of the underlying etiology.

In our study, 85.4% of cases had optimal or well-adjusted clinical outcomes during long-term treatment (39 dogs optimally adjusted; 31 well-adjusted). Only one dog died due to an adrenal crisis, indicating a generally excellent prognosis. Multinomial logistic regression analysis showed that the frequency of daily GC administration significantly impacts clinical outcomes, with the division of the daily GC dosage being associated with a reduced likelihood of achieving optimal control. Considering that prednisolone has a half-life of 12–36 h, administering it twice daily could result in drug overlap, potentially leading to increased side effects. For this reason, once-daily administration may be recommended to achieve an optimal GC therapy regimen.

While the literature often reports good clinical outcomes in dogs with HA, the incidence of iatrogenic hyperadrenocorticism remains high (14). In humans, long-term supraphysiological GC therapy can affect quality of life, increase mortality, and cause various morbidities, including higher infection risks in people, with some data available for dogs (36, 49). The physiological importance of endogenous cortisol as a biomarker for stress and welfare has also been demonstrated in other species. A study in horses, for example, showed that endogenous cortisol concentrations can vary due to environmental factors, in this case, different housing systems, and correlate with corresponding stress levels (50). This highlights the necessity of optimal GC substitution, which is essential for the well-being of dogs and their adaptation to environmental stressors. We recommend that extensive clinical judgment by the attending veterinarian must be considered a cornerstone for monitoring and adjusting the optimal GC dose. This evaluation should include detailed conservations between owner and veterinarian about potential side effects and quality of life, as well as a comprehensive physical examination and blood and urine analysis. The findings support the clinician determining the most appropriate dose.

Human guidelines suggest gradually titrating the GC dose to the lowest tolerated level and then slightly increasing it. Furthermore, they emphasize mild under-replacement is unlikely to cause an adrenal crisis, and well-defined clinical signs of underdosing can help identify the appropriate dose more quickly, potentially reducing adverse outcomes (38). This approach requires further investigation in veterinary medicine, as it may present greater risks due to the lack of verbal and physical expressions in animals. Implementing health-related quality of life (HRQoL) scores, such as the “Disease-Specific Quality of Life Questionnaire in Addison's Disease” (AddiQoL) for humans (51, 52), could help better classify and analyze clinical abnormalities in dogs with HA. While a similar HRQoL tool exists for dogs with hyperadrenocorticism (Cushing's syndrome), no comparable tool is available for dogs with HA (53).

Our study has several limitations. The main limitations of this study are its retrospective design and the focus on a single country, which constrained the data. Another limitation is the absence of a pilot study and a power analysis to determine the required sample size. Given the low prevalence of HA, the sample size was constrained, which may affect the robustness and limit the statistical power, especially of the logistic regression analysis. Our study relied on self-reported records and subjective assessments from veterinarians. Consequently, this may have introduced recall bias and inconsistencies in the treatment management of HA patients. The year 2016 was specifically chosen because DOCP (Zycortal^®^, Dechra) was approved in the European Union in November 2015. The authors assume that, as a result, many dogs were additionally treated with this medication, leading to reduced variability and increased homogeneity. Furthermore, this treatment method, involving monthly injections and regular electrolyte monitoring, is associated with a higher number of follow-ups and a greater documentation obligation, which may help minimize recall bias. Data on fluid therapy and supplementary medications administered during the acute adrenal crisis were incomplete. These factors, along with potential differences in disease pathogenesis (primary or secondary HA), might have influenced the observed clinical outcomes. The timing of electrolyte rechecks varied depending on the attending veterinarian's discretion, which introduces another source of potential bias. Ideally, standardized protocols for electrolyte monitoring and recheck intervals would have ensured greater consistency and reliability of the data. The smaller sample size in the hydrocortisone group compared to others should be noted, as it may affect the observed efficacy. The response options for hospitalization duration, electrolyte normalization, and general condition also had limitations, particularly the inability to statistically analyze responses beyond 120 h. More granular options might have revealed stronger effects between prednisolone and hydrocortisone. Therefore, the data may be slightly underestimated in this context. Another limitation of the study is that only a limited number of animals could be followed over the 2-year period. This is likely due to the fact that the majority of cases were from large veterinary clinics and referral centers, which primarily focus on the intensive treatment of inpatients rather than on follow-up examinations. Finally, while multivariable logistic regression analysis was conducted, the relatively small cohort size limited the number of associations that could be identified. A larger sample size would likely have led to more robust results and better insight into predictors of treatment outcomes. Despite these limitations, the current study offers valuable insights into the management of HA in dogs and lays a foundation for future studies investigating the benefits of hydrocortisone over prednisolone, while also identifying additional predictors to optimize treatment protocols. Additional studies are needed to evaluate the advantageous impacts of hydrocortisone. If further research confirms the superiority of hydrocortisone compared to prednisolone, the authors recommend revising the existing clinical guidelines for the management of canine HA to reflect these findings and optimize treatment strategies (54). A prospective, double-blind, placebo-controlled, outcome-based study comparing two treatment protocols (hydrocortisone vs. prednisolone) in dogs with HA is advised to avoid recall and selection bias and to ensure high validity of the results. A multicenter study would be suitable to generate an adequate study population and provide sufficient statistical power.

## 5 Conclusion

In conclusion, treatment with hydrocortisone shows promise for managing HA during initial hospitalization. However, the question of whether hydrocortisone also demonstrates superiority during the long-term course in dogs remains unanswered. Further investigations are warranted to confirm our findings during hospitalization and to evaluate the use of hydrocortisone in long-term therapy. GC therapy significantly influences clinical outcomes in dogs with HA, and the replacement therapy must be tailored to each patient's needs. Our study supports the research goal focused on the development and establishment of HRQoL scores in Addisonian dogs, which will better evaluate and manage patient welfare. This tool will assist practicing veterinarians in their decision-making process and contribute to the optimal management of afflicted dogs. Furthermore, it could serve as a cornerstone for the statistical validation of future research directions, offering a valuable basis for investigating the long-term impact of hydrocortisone therapy and other important treatment approaches.

## Data Availability

The raw data supporting the conclusions of this article will be made available by the authors, without undue reservation.
