# Supplementary material for: Comparison of prednisolone and alternative glucocorticoid dosing protocols for canine hypoadrenocorticism: insights from a survey-based study
Source: Front Vet Sci. 2025 Apr 14;12:1544750. doi: 10.3389/fvets.2025.1544750 (PMC12034680; doi:10.3389/fvets.2025.1544750)
Supplement: Supplementary file 1 [file Data_Sheet_1.pdf]

Dear colleagues,

If you are treating a dog with Addison's disease in your practice, referral center or veterinary clinic, we would be very pleased if you would take a moment to fill out this questionnaire.
Our prerequisites are that the diagnosis of hypoadrenocorticism was made between 2016-2023, and that the dog has been under treatment for 3 months or longer. In addition, the disease must have been diagnosed using an adequate test procedure (e.g. ACTH stimulation test, combination of basal cortisol and endogenous ACTH).

This survey focused about the individual glucocorticoid dosage adjustment.
Each individual case should have its own survey response. It will take you approximately 15-20 minutes to answer the questions for each dog.

The results of the survey will help to optimize the therapy for afflicted dogs, which can improve the quality and duration of life for many dogs.

If you encounter any problems, please feel free to contact me at the following email address:
christin.emming@tiho-hannover.de

The data collection for this survey is subject to the General Data Protection Regulation (GDPR), so all your personal data will be treated confidentially.

We ask that you complete this survey carefully.

We would like to thank you very much for taking the time to fill out the questionnaire.

To open the survey, please accept our privacy policy.

Part A: General questions about your patient

This section collects general data about the patient.

A1. What is the patient's gender?

- Female ☐
- Female-neutered ☐
- Masculine ☐
- Male-castrated ☐
- Unknown ☐

A2. What breed of dog is the patient?

Information: If the patient belongs to a dog breed that is not listed as an answer option, please enter it manually.

- Mixed-breed dog ☐
- Labrador Retriever ☐
- Golden Retriever ☐
- Labradoodle ☐
- Nova Scotia Duck Tolling Retriever ☐
- Poodle ☐
- Cocker Spaniel ☐
- Cairn Terrier ☐
- West Highland White Terrier ☐
- Yorkshire Terrier ☐
- Rottweiler ☐
- Great Dane ☐
- Beagle ☐
- English Springer Spaniel ☐
- Bearded Collie ☐
- No answer ☐
- Other breed: ☐

A3. What is the patient's date of birth?

Information: If your date of birth is unknown, enter 01/01/YYYY and the corresponding or estimated year of birth.

A4. What is the animal's current or last recorded weight (in kg)?

Information: Please round your body weight up or down to one decimal place.

A5. What was the weight when the diagnosis was made (in kg)?

Information: Please round your body weight up or down to one decimal place.

A6. Is the patient currently still alive?

Yes

No

Unknown

A7. Has the patient died/been euthanized due to Addison's disease?

Information: An Addisonian crisis is characterized by an acute, deteriorated general condition, dehydration, hypovolemia and collapse.

Yes, the patient died/was euthanized in the context of an acute Addisonian crisis.

Yes, the patient died/was euthanized due to the side effects of glucocorticoid therapy been.

Yes, the patient died/was euthanized due to the side effects of mineralocorticoid therapy been.

Yes, the patient died/was euthanized in connection with Addison's disease, although the exact connection is unclear.

No, the patient did not die/was not euthanized in connection with Addison's disease.

A8. When did the patient die/was euthanized?

Information: If the date is unknown, enter 01/01/YYYY and the corresponding year.

Part B: General questions about the diagnosis of Addison's disease

This section refers to the diagnosis. Only dogs that were diagnosed between January 1st, 2016 - December 31st, 2023, should be included in this study.

B1. When was the diagnosis of Addison's disease made?

Information: If the date is unknown, enter 01.MM. YYYY and the corresponding month and year. Additionally, if the month is unclear, specify 01/01/ YYYY and the corresponding year.

B2. What form of hypoadrenocorticism was diagnosed?

Primary, hyponatremic and hyperkalemic hypoadrenocorticism (disorder of the adrenal gland). ☐

Primary, eunatraemic and eukalaemic hypoadrenocorticism (disorder of the adrenal gland). ☐

Secondary glucocorticoid-deficient hypoadrenocorticism (disorder of the pituitary gland). ☐

Tertiary glucocorticoid-deficient hypoadrenocorticism (disorder of the hypothalamus). ☐

Hypoadrenocorticism without changes in sodium-potassium balance, unclear whether it is primary, secondary or tertiary hypoadrenocorticism. ☐

No answer ☐

B3. Which diagnostic test(s) was/were performed?

ACTH stimulation test ☐

Combination of basal cortisol and endogenous ACTH ☐

Combination of basal cortisol and endogenous CRH ☐

Plasma aldosterone concentration (basal or stimulated) ☐

Aldosterone-to-renin (ARR) ratio ☐

Cortisol-to-adrenocorticotropic hormone (CAR) ratio ☐

Measurement of autoantibodies (21-hydroxylase autoantibodies) ☐

No answer ☐

B4. What clinical symptoms did the patient show at the time of diagnosis?

Information: Acute Addison's crisis is characterized by an acute, deteriorated general condition, dehydration, hypovolemia and collapse.

Acute Addisonian crisis ☐

Loss of appetite ☐

Weight loss ☐

Lethargy, apathy ☐

Vomiting ☐

Diarrhea ☐

Polyuria, polydipsia ☐

Tremor ☐

Megaesophagus ☐

ECG changes ☐

No answer ☐

Miscellaneous:

B5. What changes in complete blood count, biochemistry and electrolytes were evident at this point in time?

- Absence of a stress leukogram (lymphopenia, eosinopenia, leukocytosis, monocytosis) ☐
- Hyperkalemia ☐
- Hyponatremia ☐
- Hypochloremia ☐
- Physiological electrolytes ☐
- Azotemia ☐
- Hyperphosphatemia ☐
- Hypophosphatemia ☐
- Hypercalcemia ☐
- Hypocalcemia ☐
- Hypoglycemia ☐
- Hypoalbuminemia ☐
- Elevated liver enzymes (ALT, AST) ☐
- Regenerative/ non-regenerative anemia ☐
- No answer ☐

Miscellaneous:

Part C: Questions about initial therapy for Addison's disease

This section deals with the dog's initial treatment and glucocorticoid therapy.

C1. Was the patient hospitalized?

The patient was hospitalized for at least 24 hours. ☐

The patient was hospitalized during the day until stabilization. ☐

No, the patient was cared for as an outpatient and did not require any special form of monitoring. ☐

No answer ☐

C2. How long was the hospitalization (including the patients who received day care)?

24 hours ☐

48 hours ☐

72 hours ☐

96 hours ☐

120 hours ☐

> 120 hours ☐

No answer ☐

C3. If there were corresponding electrolyte changes, when did they return to normal?

After 6 hours ☐

After 12 hours ☐

After 24 hours ☐

After 48 hours ☐

After 72 hours ☐

> 72 hours ☐

C4. After how long did the patient show an undisturbed general condition?

Information: An undisturbed general well-being includes independent walking and standing ability, physiological vital parameters and reliable and independent food and water intake.

After 12 hours ☐

After 24 hours ☐

After 48 hours ☐

After 72 hours ☐

> 72 hours ☐

C5. What initial glucocorticoid preparation did the patient receive?

Hydrocortisone ☐

Prednisone ☐

Prednisolone ☐

Methylprednisolone ☐

Dexamethasone ☐

Budesonide ☐

No glucocorticoid was administered. ☐

No answer ☐

Another unnamed glucocorticoid preparation: ☐

C6. Would you have chosen a different glucocorticoid preparation if it had been available?

Yes ☐

No ☐

C7. Which glucocorticoid would you have chosen as your medication of choice?

Hydrocortisone ☐

Prednisone ☐

Prednisolone ☐

Methylprednisolone ☐

Dexamethasone ☐

Budesonide ☐

Another unnamed glucocorticoid preparation: ☐

C8. What initial glucocorticoid dosage did you choose for the patient? Please first state the dosage in mg/kg per day (d) or, in the case of a continuous drip infusion, in mg/kg per hour (h). Then define how many administrations the dosage was divided into (once daily, twice daily, three times daily) and finally specify the type of administration (daily drip infusion (CRI), intravenous (IV), intramuscular (IM), subcutaneous (SC), peroral (PO)).

Example: 0.2 mg/kg/d - divided into twice daily administration - peroral administration (PO)
Example: 0.625 mg/kg/h – CRI – IV

Dosage in mg/kg/day or mg/kg/hour ☐☐☐ ☐☐☐☐☐☐

Into how many doses was the medication divided? ☐☐☐☐☐☐☐☐☐☐

Type of administration (IV, SC, PO) ☐☐☐☐☐☐☐☐☐☐

Part D: In the event of hospitalization or day care

This section only refers to patients who were hospitalized or cared for in day care at the beginning of the illness. The questions focus on the adjustment of the glucocorticoid therapy over the course of the inpatient stay.

D1. Was the dose of the initial glucocorticoid adjusted?

Yes, once ☐

Yes, several times ☐

No ☐

No answer ☐

D2. Was the dose of the initial glucocorticoid increased?

Yes, once ☐

Yes, several times ☐

No ☐

No answer ☐

D3. Has a change in preparation been implemented?

Yes, once ☐

Yes, several times ☐

No ☐

No answer ☐

D4. Was the glucocorticoid therapy adjusted 24 hours after admission?

Yes ☐

No ☐

No answer ☐

D5. What type of adjustment was performed?

Dose increase ☐

Dose reduction ☐

Change of preparation ☐

Changing of the frequency of administration (example: from twice daily to once daily) ☐

Discontinuation of the medication ☐

No answer ☐

D6. Which drug did you choose when adjusting the glucocorticoid therapy?

Hydrocortisone ☐

Prednisone ☐

Prednisolone ☐

Methylprednisolone ☐

Dexamethasone ☐

Budesonide ☐

Another unnamed glucocorticoid preparation: ☐

D7. Which adjusted glucocorticoid dosage did you choose for the patient after 24 hours? Please first state the dosage in mg/kg per day (d) or, in the case of a continuous drip infusion, in mg/kg per hour (h). Then define how many administrations the dosage was divided into (once daily, twice daily, three times daily) and finally specify the type of administration (daily drip infusion (CRI), intravenous (IV), intramuscular (IM), subcutaneous (SC), peroral (PO)).

Example: 0.2mg/kg/d - divided into twice daily administration - PO
Example: 0.625 mg/kg/h - CRI - IV

Dosage in mg/kg/day or mg/kg/hour

Into how many doses was the medication divided? ☐☐☐☐☐☐☐☐☐☐

Type of administration (IV, SC, PO) ☐☐☐☐☐☐☐☐☐☐

D8. Why was therapy modified 24 hours after admission?

Clinical examples suggestive of a glucocorticoid overdose: polyuria and polydipsia/ polyphagia/ panting/ weight gain/ depression/ listlessness. Clinical examples suggestive of glucocorticoid underdosage: anorexia/ weight loss/ vomiting/ diarrhea/ weakness/ fatigue/ stress intolerance.

Good response to initial therapy (rapid improvement in vital and laboratory parameters). ☐

Moderate response to initial therapy (moderate improvement in vital and laboratory parameters). ☐

No response to initial therapy (no improvement in vital and laboratory parameters). ☐

Adjustment due to expected potential side effects. ☐

Regarding the impairment of one or more previously diagnosed diseases. ☐

Regarding the interaction with other medications that the patient is taking. ☐

Occurrence of side effects suggesting a glucocorticoid overdose. ☐

Occurrence of clinical signs suggesting glucocorticoid deficiency. ☐

Preparation for home care. ☐

No answer ☐

Miscellaneous: ☐

D9. How were the clinical symptoms and general condition affected by the aforementioned modification?

Improvement ☐

Deterioration ☐

Consistent ☐

D10. If there are existing electrolyte changes (hyponatremia, hyperkalemia), how were these affected by the aforementioned modification?

Improvement ☐

Deterioration ☐

Consistent ☐

D11. Was the glucocorticoid therapy adjusted 48 hours after admission?

Yes ☐

No ☐

D12. What type of adjustment was performed?

Dose increase ☐

Dose reduction ☐

Change of preparation. ☐

Changing of the frequency of administration (example: from twice daily to once daily). ☐

Discontinuation of the medication ☐

No answer ☐

D13. Which drug did you choose when adjusting the glucocorticoid therapy?

Hydrocortisone ☐

Prednisone ☐

Prednisolone ☐

Methylprednisolone ☐

Dexamethasone ☐

Budesonide ☐

Another unnamed glucocorticoid preparation: ☐

D14. Which adjusted glucocorticoid dosage did you choose for the patient after 48 hours? Please first state the dosage in mg/kg per day (d) or, in the case of a continuous drip infusion, in mg/kg per hour (h). Then define how many administrations the dosage was divided into (once daily, twice daily, three times daily) and finally specify the type of administration (daily drip infusion (CRI), intravenous (IV), intramuscular (IM), subcutaneous (SC), peroral (PO)).

Example: 0.2mg/kg/d - divided into twice daily administration - PO
Example: 0.625 mg/kg/h - CRI - IV

Dosage in mg/kg/day or mg/kg/hour

Into how many doses was the medication divided?

Type of administration (IV, SC, PO)

D15. Why was therapy modified 48 hours after admission?

Clinical examples suggestive of a glucocorticoid overdose: polyuria and polydipsia/ polyphagia/ panting/ weight gain/ depression/ listlessness. Clinical examples suggestive of glucocorticoid underdosage: anorexia/ weight loss/ vomiting/ diarrhea/ weakness/ fatigue/ stress intolerance.

Good response to initial therapy (rapid improvement in vital and laboratory parameters). ☐

Moderate response to initial therapy (moderate improvement in vital and laboratory parameters). ☐

No response to initial therapy (no improvement in vital and laboratory parameters). ☐

Adjustment due to expected potential side effects ☐

Regarding the impairment of one or more previously diagnosed diseases. ☐

Regarding the interaction with other medications that the patient is taking. ☐

Occurrence of side effects suggesting a glucocorticoid overdose. ☐

Occurrence of clinical signs suggesting glucocorticoid deficiency ☐

Preparation for home care. ☐

No answer ☐

Miscellaneous: ☐

D16. How were the clinical symptoms and general condition affected by the aforementioned modification?

Improvement ☐

Deterioration ☐

Consistent ☐

D17. If there are existing electrolyte changes (hyponatremia, hyperkalemia), how were these affected by the aforementioned modification?

Improvement ☐

Deterioration ☐

Consistent ☐

D18. Was the glucocorticoid therapy adjusted 72 hours after admission?

Yes ☐

No ☐

D19. What type of adjustment was performed?

Dose increase ☐

Dose reduction ☐

Change of preparation. ☐

Changing of the frequency of administration (example: from twice daily to once daily). ☐

Discontinuation of the medication ☐

No answer ☐

D20. Which drug did you choose when adjusting the glucocorticoid therapy?

Hydrocortisone ☐

Prednisone ☐

Prednisolone ☐

Methylprednisolone ☐

Dexamethasone ☐

Budesonide ☐

Another unnamed glucocorticoid preparation: ☐

D21. Which adjusted glucocorticoid dosage did you choose for the patient after 72 hours? Please first state the dosage in mg/kg per day (d) or, in the case of a continuous drip infusion, in mg/kg per hour (h). Then define how many administrations the dosage was divided into (once daily, twice daily, three times daily) and finally specify the type of administration (daily drip infusion (CRI), intravenous (IV), intramuscular (IM), subcutaneous (SC), peroral (PO)).

Example: 0.2mg/kg/d - divided into twice daily administration - PO
Example: 0.625 mg/kg/h - CRI - IV

Dosage in mg/kg/day or mg/kg/hour

Into how many doses was the medication divided?

Type of administration (IV, SC, PO) ☐☐☐☐☐☐☐☐☐☐

D22. Why was therapy modified 72 hours after admission?

Clinical examples suggestive of a glucocorticoid overdose: polyuria and polydipsia/ polyphagia/ panting/ weight gain/ depression/ listlessness. Clinical examples suggestive of glucocorticoid underdosage: anorexia/ weight loss/ vomiting/ diarrhea/ weakness/ fatigue/ stress intolerance.

Good response to initial therapy (rapid improvement in vital and laboratory parameters). ☐

Moderate response to initial therapy (moderate improvement in vital and laboratory parameters). ☐

No response to initial therapy (no improvement in vital and laboratory parameters). ☐

Adjustment due to expected potential side effects. ☐

Regarding the impairment of one or more previously diagnosed diseases. ☐

Regarding the interaction with other medications that the patient is taking. ☐

Occurrence of side effects suggesting a glucocorticoid overdose. ☐

Occurrence of clinical signs suggesting glucocorticoid deficiency. ☐

Preparation for home care. ☐

No answer ☐

Miscellaneous: ☐

D23. How were the clinical symptoms and general condition affected by the aforementioned modification?

Improvement ☐

Deterioration ☐

Consistent ☐

D24. If there are existing electrolyte changes (hyponatremia, hyperkalemia), how were these affected by the aforementioned modification?

Improvement ☐

Deterioration ☐

Consistent ☐

D25. Which medication did you choose when the animal was discharged?

- Hydrocortisone ☐
- Prednisone ☐
- Prednisolone ☐
- Methylprednisolone ☐
- Dexamethasone ☐
- Budesonide ☐
- No glucocorticoid was administered. ☐
- No answer ☐
- ☐ Another unnamed glucocorticoid preparation: ______

D26. What glucocorticoid dosage did you choose when the patient was discharged? Please enter the dosage in mg/kg per day (d) first. Then define how many doses the dosage was divided into (once daily, twice daily, three times daily).

Example: 0.2mg/kg/d — divided into twice daily doses

Dosage in mg/kg/day ☐☐☐☐☐☐☐☐☐☐

Into how many doses was the medication divided? ☐☐☐☐☐☐☐☐☐☐

Part E: Questions about initial mineralocorticoid therapy

This section addresses the patient's initial mineralocorticoid therapy.

E1. When did the patient receive the first mineralocorticoid supplementation?

Simultaneously alongside initial glucocorticoid supplementation. ☐

When the diagnosis of hypoadrenocorticism was confirmed. ☐

When the patient is discharged to home care. ☐

After 10-14 days ☐

The patient is not receiving mineralocorticoid therapy. ☐

No answer ☐

Other time: ☐

E2. What initial mineralocorticoid medication did the patient receive?

Information: Fludrocortisone, for example, is Astonin® H 0.1 mg tablets (Merck Serono GmbH), while deoxycorticosterone pivalate (DOCP) is Zycortal® 25mg/ml depot injection suspension (Dechra).

Fludrocortisone ☐

Deoxycorticosterone pivalate (DOCP) ☐

Initially Fludrocortisone and, if the diagnosis was confirmed, switching to DOCP. ☐

No answer ☐

E3. What initial Fludrocortisone dosage did you choose for the patient? Please enter the dosage in mg/kg per day (d) first. Then define how many doses the dosage was divided into (once daily, twice daily, three times daily).

Example: 0.02mg/kg/day — divided into twice daily doses

Dosage in mg/kg/day

Into how many doses was the medication divided?

E4. What initial DOCP dosage did you choose for the patient? Please indicate the dosage in mg/kg. Then enter how many days after you gave the second injection.

Example: 1.5m/kg — 28 days

Dosage in mg/kg

When was the second injection administered?

Part F: Questions about adjusting glucocorticoid therapy as the disease progresses

This question section focuses on glucocorticoid therapy modification as the disease progresses. We are interested in adjusting therapy 4 weeks, 3 months, 6 months, 1 year after diagnosis and the current therapy.

F1. Was the glucocorticoid dosage reduced during the course of the disease?

Yes, once ☐

Yes, several times ☐

No ☐

Continued care provided by another veterinarian. ☐

F2. Was the glucocorticoid dosage increased during the course of the disease?

Yes, once ☐

Yes, several times ☐

No ☐

Continued care provided by another veterinarian. ☐

F3. Was the type of preparation changed during the course of therapy?

Example: Switching from prednisolone to hydrocortisone

Yes, once ☐

Yes, several times ☐

No ☐

Continued care provided by another veterinarian. ☐

F4. Was the glucocorticoid therapy adjusted 4 weeks after the diagnosis?

Yes ☐

No ☐

Continued care provided by another veterinarian. ☐

The patient died. ☐

F5. What type of adjustment was performed after 4 weeks?

Dose increase ☐

Dose reduction ☐

Change of preparation. ☐

Changing of the frequency of administration (example: from twice daily to once daily). ☐

Discontinuation of the medication ☐

☐

No answer

F6. Which drug did you choose after <u>4 weeks</u> when adjusting the glucocorticoid therapy?

Hydrocortisone

Prednisone

Prednisolone

Methylprednisolone

Dexamethasone

Budesonide

Another unnamed glucocorticoid preparation:

F7. What glucocorticoid dosage did you choose when adjusting the patient after 4 weeks? Please enter the dosage in mg/kg per day (d) first. Then define how many doses the dosage was divided into (once daily, twice daily, three times daily).

Example: 0.1 mg/kg/d — once daily administration

Dosage in mg/kg/day

Into how many doses was the medication divided?

F8. Why was therapy modified 4 weeks after diagnosis?

Clinical signs indicating a glucocorticoid overdose: polyuria and polydipsia/ polyphagia/ panting/ weight gain/ depression/ listlessness/ hair loss/dry, firm skin/ muscle loss/ increased abdominal circumference. Clinical signs suggestive of glucocorticoid underdosage: anorexia/ weight loss/ vomiting/ diarrhea/ abdominal pain/ weakness/ fatigue/ stress intolerance

Finding the optimal dose (lowest effective dose).

Adjustment due to expected potential side effects.

Moderate response to therapy (moderate improvement in vital and laboratory parameters).

No response to therapy (no improvement in vital and laboratory parameters).

Regarding the impairment of one or more previously diagnosed diseases.

Regarding the interaction with other medications that the patient is taking.

Occurrence of side effects suggesting a glucocorticoid overdose.

Occurrence of clinical signs suggesting glucocorticoid deficiency.

No answer

Miscellaneous:

F9. How did the clinical symptoms and general condition and affect the aforementioned modification?

Improvement ☐

Deterioration ☐

Consistent ☐

F10. Was the glucocorticoid therapy adjusted 3 months after diagnosis?

Yes ☐

No ☐

Continued care provided by another veterinarian. ☐

The patient died. ☐

F11. What type of adjustment was performed after 3 months?

Dose increase ☐

Dose reduction ☐

Change of preparation. ☐

Changing of the frequency of administration (example: from twice daily to once daily). ☐

Discontinuation of the medication ☐

No answer ☐

F12. Which drug did you choose after 3 months when adjusting the glucocorticoid therapy?

Hydrocortisone ☐

Prednisone ☐

Prednisolone ☐

Methylprednisolone ☐

Dexamethasone ☐

Budesonide ☐

☐ Another unnamed glucocorticoid preparation:

F13. What glucocorticoid dosage did you choose when adjusting the patient after <u>3 months</u>? Please enter the dosage in mg/kg per day (d) first. Then define how many doses the dosage was divided into (once daily, twice daily, three times daily).

Example: 0.1 mg/kg/d — once daily administration

Dosage in mg/kg/day

Into how many doses was the medication divided?

F14. Why was therapy modified <u>3 months</u> after diagnosis?

Clinical signs indicating a glucocorticoid overdose: polyuria and polydipsia/ polyphagia/ panting/ weight gain/ depression/ listlessness/ hair loss/dry, firm skin/ muscle loss/ increased abdominal circumference. Clinical signs suggestive of glucocorticoid underdosage: anorexia/ weight loss/ vomiting/ diarrhea/ abdominal pain/ weakness/ fatigue/ stress intolerance.

Finding the optimal dose (lowest effective dose).

Adjustment due to expected potential side effects.

Moderate response to therapy (moderate improvement in vital and laboratory parameters).

No response to therapy (no improvement in vital and laboratory parameters).

Regarding the impairment of one or more previously diagnosed diseases.

Regarding the interaction with other medications that the patient is taking.

Occurrence of side effects suggesting a glucocorticoid overdose.

Occurrence of clinical signs suggesting glucocorticoid deficiency.

No answer

Miscellaneous

F15. How did the clinical symptoms and general condition and affect the aforementioned modification?

Improvement ☐

Deterioration ☐

Consistent ☐

F16. Was the glucocorticoid therapy adjusted <u>6 months</u> after diagnosis?

Yes ☐

No ☐

Continued care provided by another veterinarian. ☐

The patient died. ☐

F17. What type of adjustment was performed after <u>6 months</u>?

Dose increase ☐

Dose reduction ☐

Change of preparation. ☐

Changing of the frequency of administration (example: from twice daily to once daily). ☐

Discontinuation of the medication ☐

No answer ☐

F18. Which drug did you choose after <u>6 months</u> when adjusting the glucocorticoid therapy?

Hydrocortisone ☐

Prednisone ☐

Prednisolone ☐

Methylprednisolone ☐

Dexamethasone ☐

Budesonide ☐

Another unnamed glucocorticoid preparation: ☐

F19. What glucocorticoid dosage did you choose when adjusting the patient after 6 moths? Please enter the dosage in mg/kg per day (d) first. Then define how many doses the dosage was divided into (once daily, twice daily, three times daily).

Example: 0.1 mg/kg/d — once daily administration

Dosage in mg/kg/day

Into how many doses was the medication divided?

F20. Why was therapy modified 6 months after diagnosis?

Clinical signs indicating a glucocorticoid overdose: polyuria and polydipsia/ polyphagia/ panting/ weight gain/ depression/ listlessness/ hair loss/ dry, firm skin/ muscle loss/ increased abdominal circumference. Clinical signs suggestive of glucocorticoid underdosage: anorexia/ weight loss/ vomiting/ diarrhea/ abdominal pain/ weakness/ fatigue/ stress intolerance.

Finding the optimal dose (lowest effective dose).

Adjustment due to expected potential side effects.

Moderate response to therapy (moderate improvement in vital and laboratory parameters).

No response to therapy (no improvement in vital and laboratory parameters).

Regarding the impairment of one or more previously diagnosed diseases.

Regarding the interaction with other medications that the patient is taking.

Occurrence of side effects suggesting a glucocorticoid overdose.

Occurrence of clinical signs suggesting glucocorticoid deficiency.

No answer

Miscellaneous:

F21. How did the clinical symptoms and general condition and affect the aforementioned modification?

Improvement

Deterioration

Consistent

F22. Was the glucocorticoid therapy adjusted 1 year after diagnosis?

- Yes ☐
- No ☐
- Continued care provided by another veterinarian. ☐
- The patient died. ☐

F23. What type of adjustment was performed after 1 year?

- Dose increase ☐
- Dose reduction ☐
- Change of preparation. ☐
- Changing of the frequency of administration (example: from twice daily to once daily). ☐
- Discontinuation of the medication ☐
- No answer ☐

F24. Which drug did you choose after 1 year when adjusting the glucocorticoid therapy?

- Hydrocortisone ☐
- Prednisone ☐
- Prednisolone ☐
- Methylprednisolone ☐
- Dexamethasone ☐
- budesonide ☐
- Another unnamed glucocorticoid preparation: ☐

F25. What glucocorticoid dosage did you choose when adjusting the patient after 1 year? Please enter the dosage in mg/kg per day (d) first. Then define how many doses the dosage was divided into (once daily, twice daily, three times daily).

Example: 0.1 mg/kg/d — once daily administration

Dosage in mg/kg/day

Into how many doses was the medication divided?

F26. Why was therapy modified 1 year after diagnosis?

Clinical signs indicating a glucocorticoid overdose: polyuria and polydipsia/ polyphagia/ panting/ weight gain/ depression/ listlessness/ hair loss/dry, firm skin/ muscle loss/ increased abdominal circumference. Clinical signs suggestive of glucocorticoid underdosage: anorexia/ weight loss/ vomiting/ diarrhea/ abdominal pain/ weakness/ fatigue/ stress intolerance.

- Finding the optimal dose (lowest effective dose). ☐
- Adjustment due to expected potential side effects. ☐
- Moderate response to therapy (moderate improvement in vital and laboratory parameters). ☐
- No response to therapy (no improvement in vital and laboratory parameters). ☐
- Regarding the impairment of one or more previously diagnosed diseases. ☐
- Regarding the interaction with other medications that the patient is taking. ☐
- Occurrence of side effects suggesting a glucocorticoid overdose. ☐
- Occurrence of clinical signs suggesting glucocorticoid deficiency. ☐
- No answer ☐

Miscellaneous:

F27. How did the clinical symptoms and general condition and affect the aforementioned modification?

- Improvement ☐
- Deterioration ☐
- Consistent ☐

F28. Was the glucocorticoid therapy adjusted 2 years after diagnosis?

- Yes ☐
- No ☐
- Continued care provided by another veterinarian. ☐
- The patient died. ☐

F29. What type of adjustment was performed after 2 years?

Dose increase ☐

Dose reduction ☐

Change of preparation. ☐

Changing of the frequency of administration (example: from twice daily to once daily). ☐

Discontinuation of the medication ☐

No answer ☐

F30. Which drug did you choose after 2 years when adjusting the glucocorticoid therapy?

Hydrocortisone ☐

Prednisone ☐

Prednisolone ☐

Methylprednisolone ☐

Dexamethasone ☐

Budesonide ☐

Another unnamed glucocorticoid preparation: ☐

F31. What glucocorticoid dosage did you choose when adjusting the patient after 2 years? Please enter the dosage in mg/kg per day (d) first. Then define how many doses the dosage was divided into (once daily, twice daily, three times daily).

Example: 0.1 mg/kg/d — once daily administration

Dosage in mg/kg/day

Into how many doses was the medication divided?

F32. Why was therapy modified 2 years after diagnosis?

Clinical signs indicating a glucocorticoid overdose: polyuria and polydipsia/ polyphagia/ panting/ weight gain/ depression/ listlessness/ hair loss/dry, firm skin/ muscle loss/ increased abdominal circumference. Clinical signs suggestive of glucocorticoid underdosage: anorexia/ weight loss/ vomiting/ diarrhea/ abdominal pain/ weakness/ fatigue/ stress intolerance.

Finding the optimal dose (lowest effective dose). ☐

Adjustment due to expected potential side effects. ☐

Moderate response to therapy (moderate improvement in vital and laboratory parameters). ☐

No response to therapy (no improvement in vital and laboratory parameters). ☐

Regarding the impairment of one or more previously diagnosed diseases. ☐

Regarding the interaction with other medications that the patient is taking. ☐

Occurrence of side effects suggesting a glucocorticoid overdose. ☐

Occurrence of clinical signs suggesting glucocorticoid deficiency. ☐

No answer ☐

Miscellaneous:

F33. How did the clinical symptoms and general condition and affect the aforementioned modification?

Improvement ☐

Deterioration ☐

Consistent ☐

Part G: Questions about current or most recently known glucocorticoid therapy

This part is about your patient's current glucocorticoid dosage. If the dog has already died, state the last known glucocorticoid therapy.

G1. What is the current or last known glucocorticoid medication that the patient is receiving or has received?

Information: If the patient has already died, please select the last medication given.

- Hydrocortisone ☐
- Prednisone ☐
- Prednisolone ☐
- Methylprednisolone ☐
- Dexamethasone ☐
- Budesonide ☐
- Continued care provided by another veterinarian. ☐
- The glucocorticoid medication was discontinued. ☐
- Another unnamed glucocorticoid preparation: ☐

G2. What <u>current or last known</u> dosage is the patient receiving or receiving? Please enter the dosage in mg/kg per day (d) first. Then define how many doses the dosage is or was divided into (once daily, twice daily, three times daily).

Information: If the patient has already died, please select the last dosage given.
Example: 0.1 mg/kg/d — once daily administration

Dosage in mg/kg/day

Into how many doses was the medication divided?

G3. In your opinion, is or was Addison's disease optimally controlled with regard to the current or most recently known glucocorticoid dosage?

Information: An optimal setting is measured based on general well-being, vital parameters and the presence of side effects that suggest a deficiency or overdose.

- The dog is or was optimally adjusted. ☐
- The dog is or was well adjusted (minor signs of symptoms). ☐
- The dog is or was moderately adjusted (some signs of symptoms). ☐
- The dog is or was poorly adjusted (excess of symptoms). ☐
- The dog is or was very poorly adjusted (The treatment wasn’t improving the disease). ☐

No statement is possible because the dog also has other illnesses that worsen the clinical picture. ☐

G4. Do you use or have you used basal ACTH testing to optimize glucocorticoid replacement therapy?"

Yes, the test proved helpful. ☐

Yes, but the test was not useful. ☐

No ☐

Part H: Questions about your patient's glucocorticoid dosage in stressful situations

In this section we would like to hear from you how to manage your patient's glucocorticoid therapy in stressful situations.

H1. Do you recommend that the patient owner briefly increase the glucocorticoid dosage in stressful situations?

Yes, in any kind of stressful situation (vacation, visit to the vet, heat). ☐

Yes, only in an exceptionally stressful situation (New Year's Eve, fireworks). ☐

No, I do not recommend increasing the glucocorticoid dosage in a stressful situation. ☐

H2. If you recommend increasing the glucocorticoid dosage in stressful situations, what is the average increase?

10% ☐

25% ☐

50% ☐

75% ☐

100% ☐

Other information: ☐

H3. What is the recommended average duration for increasing the glucocorticoid dosage in the case of a stressful situation?

< 2 days ☐

2 days ☐

3 days ☐

4 days ☐

7 days ☐

Another period: ☐

H4. On average, how long does it take to reduce the increased glucocorticoid dosage after a stressful situation?

- < 2 days ☐
- 2 days ☐
- 3 days ☐
- 4 days ☐
- 7 days ☐
- ☐ Another period:

Part I: Questions about adjusting mineralocorticoid therapy as the disease progresses

This part is about adjusting mineralocorticoid therapy as the disease progresses.

I1. Was the mineralocorticoid supplementation adjusted during the course of the disease?

- Yes, once ☐
- Yes, several times ☐
- No ☐
- The medication was discontinued. ☐
- The patient does not receive mineralocorticoid supplementation. ☐
- Continued care provided by another veterinarian. ☐

I2. Was mineralocorticoid supplementation reduced during the course of the disease?

- Yes, once ☐
- Yes, several times ☐
- No ☐

I3. Was mineralocorticoid supplementation increased during the course of the disease?

- Yes, once ☐
- Yes, several times ☐
- ☐

No

I4. Was the type of preparation changed during the course of the disease?

Information: Fludrocortisone, for example, is Astonin® H 0.1 mg tablets (Merck Serono GmbH), while deoxycorticosterone pivalate (DOCP) is Zycortal® 25mg/ml depot injection suspension (Dechra).

Yes, from DOCP to Fludrocortisone. ☐

Yes, from fludrocortisone to DOCP. ☐

No, the patient continues to receive Fludrocortisone. ☐

No, the patient continues to receive DOCP. ☐

I5. Why was the type of preparation changed during the course of the disease?

Persistent electrolyte abnormalities that indicate mineralocorticoid underdosage (hyperkalemia, hyponatremia). ☐

Persistent electrolyte abnormalities that indicate a mineralocorticoid overdose (hypokalemia, hypernatremia). ☐

Clinical abnormalities ☐

Drug intolerance ☐

No answer ☐

Miscellaneous: ☐

I6. What is the current or last known Fludrocortisone dosage for the patient? Please enter the dosage in mg/kg per day (d) first. Then define how many doses the dosage is or was divided into (once daily, twice daily, three times daily).

Example: 0.02mg/kg/day — divided into twice daily doses

Dosage in mg/kg/day

Into how many doses was the medication divided?

I7. What is the current or last known DOCP dosage for the patient? Please indicate the dosage in mg/kg. Then enter after how many days you give or gave the next injection.

Example: 1.5m/kg — 28 days

Dosage in mg/kg

Next injection after how many days?

Part J: Questions about other diseases and comorbidities of the patient

The last section of questions deals with other diseases that the patient may suffer from or have suffered from. We only want to focus on endocrinological, immune-mediated and/or inflammatory diseases.

J1. Does the patient suffer from one or more other endocrinological, immune-mediated or inflammatory diseases?

Information: endocrinological disease e.g. hypothyroidism; Immune-mediated disease e.g. primary immune-mediated anemia; inflammatory disease e.g. pancreatitis

- The patient has or had another illness. ☐
- The patient has or had two other illnesses. ☐
- The patient has or had three other illnesses. ☐
- The patient has or had > three other illnesses. ☐
- No, the patient does not suffer from or did not suffer from any other illness. ☐
- Continued care provided by another veterinarian. ☐

J2. What is the first disease the patient suffers from or suffered from?

- Hypothyroidism ☐
- Hypoparathyroidism ☐
- Hyperparathyroidism ☐
- Diabetes mellitus ☐
- Diabetes insipidus ☐
- Immune-mediated anemia ☐
- Immune-mediated thrombocytopenia ☐
- Azoospermia ☐
- Glomerulopathy ☐
- Pancreatitis ☐
- Food-responsive enteropathy ☐
- Immunosuppressant-responsive enteropathy ☐
- Other disease: ☐

J3. When was the first disease diagnosed?

Before the diagnosis of hypoadrenocorticism. ☐

At the same time as the diagnosis of hypoadrenocorticism. ☐

After the diagnosis of hypoadrenocorticism. ☐

J4. Was the first disease successfully treated, or does it still exist?

The disease was successfully treated, and the patient is no longer receiving or receiving medication. ☐

The disease is or was under treatment and the patient is or was well adjusted. ☐

The disease is or was under treatment and the patient has or had minor symptoms. ☐

The disease is or was under treatment and the patient has or had moderate symptoms. ☐

The disease is or was under treatment and the patient has or had severe symptoms ☐

The patient died of the disease or was euthanized because of it. ☐

J5. Which medication(s) is the patient receiving for their first disease?

Information: Please enter the medication(s) manually

J6. What is the second disease the patient suffers from or suffered from?

Hypothyroidism ☐

Hypoparathyroidism ☐

Hyperparathyroidism ☐

Diabetes mellitus ☐

Diabetes insipidus ☐

Immune-mediated anemia ☐

Immune-mediated thrombocytopenia ☐

Azoospermia ☐

Glomerulopathy ☐

Pancreatitis ☐

Food-responsive enteropathy ☐

Immunosuppressant-responsive enteropathy ☐

Other disease:

J7. When was the second disease diagnosed?

Before the diagnosis of hypoadrenocorticism. ☐

At the same time as the diagnosis of hypoadrenocorticism. ☐

After the diagnosis of hypoadrenocorticism. ☐

J8. Was the second disease successfully treated, or does it still exist?

The disease was successfully treated, and the patient is no longer receiving or receiving medication. ☐

The disease is or was under treatment and the patient is or was well adjusted. ☐

The disease is or was under treatment and the patient has or had minor symptoms. ☐

The disease is or was under treatment and the patient has or had moderate symptoms. ☐

The disease is or was under treatment and the patient has or had severe symptoms. ☐

The patient died of the disease or was euthanized because of it. ☐

J9. Which medication(s) is the patient receiving for their second disease?

Information: Please enter the medication(s) manually

J10. What is the third disease the patient suffers from or suffered from?

Hypothyroidism ☐

Hypoparathyroidism ☐

Hyperparathyroidism ☐

Diabetes mellitus ☐

Diabetes insipidus ☐

Immune-mediated anemia ☐

☐

Immune-mediated thrombocytopenia

Azoospermia ☐

Glomerulopathy ☐

Pancreatitis ☐

Food-responsive enteropathy ☐

Immunosuppressant-responsive enteropathy ☐

Other disease: ☐

J11. When was the <u>third disease</u> diagnosed?

Before the diagnosis of hypoadrenocorticism. ☐

At the same time as the diagnosis of hypoadrenocorticism. ☐

After the diagnosis of hypoadrenocorticism. ☐

J12. Was the <u>third disease</u> successfully treated, or does it still exist?

The disease was successfully treated, and the patient is no longer receiving or receiving medication. ☐

The disease is or was under treatment and the patient is or was well adjusted. ☐

The disease is or was under treatment and the patient has or had minor symptoms. ☐

The disease is or was under treatment and the patient has or had moderate symptoms. ☐

The disease is or was under treatment and the patient has or had severe symptoms. ☐

The patient died of the disease or was euthanized because of it. ☐

J13. Which medication(s) is the patient receiving for their <u>third disease</u>?

Information: Please enter the medication(s) manually.

J14. What is the <u>fourth disease</u> the patient suffers from or suffered from?

Information: You have indicated that the patient suffers from more than three additional endocrinological, immune-mediated and/or inflammatory diseases. Please describe the four diseases with the highest priority.

- Hypothyroidism ☐
- Hypoparathyroidism ☐
- Hyperparathyroidism ☐
- Diabetes mellitus ☐
- Diabetes insipidus ☐
- Immune-mediated anemia ☐
- Immune-mediated thrombocytopenia ☐
- Azoospermia ☐
- Glomerulopathy ☐
- Pancreatitis ☐
- Food-responsive enteropathy ☐
- Immunosuppressant-responsive enteropathy ☐
- Other disease: ☐

J15. When was the <u>fourth disease</u> diagnosed?

- Before the diagnosis of hypoadrenocorticism. ☐
- At the same time as the diagnosis of hypoadrenocorticism. ☐
- After the diagnosis of hypoadrenocorticism. ☐

J16. Was the <u>fourth disease</u> successfully treated, or does it still exist?

- The disease was successfully treated, and the patient is no longer receiving or receiving medication ☐
- The disease is or was under treatment and the patient is or was well adjusted. ☐
- The disease is or was under treatment and the patient has or had minor symptoms. ☐
- The disease is or was under treatment and the patient has or had moderate symptoms. ☐
- The disease is or was under treatment and the patient has or had severe symptoms. ☐
- The patient died of the disease or was euthanized because of it. ☐

J17. Which medication(s) is the patient receiving for their <u>fourth disease</u>?

Information: Please enter the medication(s) manually.

Thank you for participating in the survey!
