# Supplementary material for: Comparison of prednisolone and alternative glucocorticoid dosing protocols for canine hypoadrenocorticism: insights from a survey-based study
Source: Front Vet Sci. 2025 Apr 14;12:1544750. doi: 10.3389/fvets.2025.1544750 (PMC12034680; doi:10.3389/fvets.2025.1544750)
Supplement: Supplementary file 2 [file Data_Sheet_2.pdf]

Sehr geehrte Kollegen/innen,

sollten Sie in Ihrer Praxis, in Ihrem Überweisungszentrum oder in Ihrer Tierklinik einen Hund mit Morbus Addison therapieren, würden wir uns sehr freuen, wenn Sie sich kurz Zeit nehmen, um diesen Fragebogen auszufüllen.

Voraussetzung ist, dass die Diagnose Hypoadrenokortizismus zwischen 2016-2023 gestellt wurde und der Hund seit 3 Monaten oder länger in Behandlung ist. Darüber hinaus muss die Erkrankung mithilfe eines adäquaten Testverfahrens diagnostiziert worden sein (z.B. ACTH-Stimmulationstest, Kombination aus basalem Cortisol und endogenem ACTH). Die Befragung nach der individuellen Glukokortikoid-Dosisanpassung im Verlaufe der Erkrankung, beschränkt sich auf die ersten zwei Jahre nach Therapiestart. Für die Beantwortung der Fragen werden Sie ungefähr 15-20 Minuten benötigen.

Ihre Erkenntnisse können dazu beitragen die Therapie von erkrankten Hunden zu optimieren, wodurch die Lebensqualität und -dauer von vielen Hunden verbessert werden kann. Bei Auftreten von Problemen können Sie sich gerne an die folgende E-Mail-Adresse wenden: christin.emming@tiho-hannover.de

Die Datenerhebung dieser Umfrage unterliegt der Datenschutzgrundverordnung (DSGVO), somit werden alle Ihre persönlichen Daten vertraulich behandelt.

Wir bitten Sie, diese Umfrage gewissenhaft auszufüllen.

Wir bedanken uns recht herzlich, dass Sie sich die Mühe machen, den Fragebogen auszufüllen.

Um die Umfrage zu öffnen, akzeptieren Sie bitte unsere Datenschutzerklärung.

Teil A: Allgemeine Fragen zu Ihrem Patienten

In diesem Abschnitt werden allgemeine Daten über den Patienten erhoben.

A1. Welches Geschlecht hat der Patient?

- Weiblich ☐
- Weiblich-kastriert ☐
- Männlich ☐
- Männlich-kastriert ☐
- Unbekannt ☐

A2. Zu welcher Hunderasse gehört der Patient?

Info: Sollte der Patient einer Hunderasse angehören, welche nicht als Antwortoption aufgeführt ist, so geben Sie diese bitte manuell ein

- Mischling ☐
- Labrador Retriever ☐
- Golden Retriever ☐
- Labradoodle ☐
- Nova Scotia Duck Tolling Retriever ☐
- Pudel ☐
- Cockerspaniel ☐
- Cairn Terrier ☐
- West Highland White Terrier ☐
- Yorkshire Terrier ☐
- Rottweiler ☐
- Dogge ☐
- Beagle ☐
- English Springer Spaniel ☐
- Bearded Collie ☐
- Keine Antwort ☐
- Andere Rasse: ☐

A3. Wie lautet das Geburtsdatum des Patienten?

Info: Bei unbekanntem Geburtsdatum, tragen Sie 01.01.JJJJ und das entsprechende oder geschätzte Geburtsjahr ein.

A4. Wie lautet das aktuelle Gewicht des Tieres (in kg Körpergewicht)?

Info: Bitte runden Sie das Körpergewicht auf eine Nachkommastelle auf bzw. ab.

A5. Wie lautete das Gewicht bei der Diagnosestellung (in kg Körpergewicht)?

Info: Bitte runden Sie das Körpergewicht auf eine Nachkommastelle auf bzw. ab.

A6. Ist der Patient aktuell noch am Leben?

Ja ☐

Nein ☐

Unbekannt ☐

A7. Ist der Patient im Zusammenhang mit dem Morbus Addison verstorben/ eingeschläfert worden?

Info: Eine Addison-Krise ist gekennzeichnet mit einem akut auftretenden, verschlechterten Allgemeinbefinden, Dehydratation, Hypovolämie und Kollaps.

Ja, der Patient ist im Zusammenhang mit einer akuten Addison-Krise verstorben/eingeschläfert worden. ☐

Ja, der Patient ist durch die Nebenwirkungen der Glukokortikoid-Therapie verstorben/eingeschläfert worden. ☐

Ja, der Patient ist durch die Nebenwirkungen der Mineralokortikoid-Therapie verstorben/eingeschläfert worden. ☐

Ja, der Patient ist im Zusammenhang mit dem Morbus Addison verstorben/eingeschläfert worden, allerdings ist der genaue Zusammenhang unklar. ☐

Nein, der Patient ist nicht im Zusammenhang mit dem Morbus Addison verstorben/eingeschläfert worden. ☐

A8. Wann ist der Patient verstorben oder eingeschläfert worden?

Info: Bei unbekanntem Datum, tragen Sie 01.01.JJJJ und das entsprechende Jahr ein.

Teil B: Allgemeine Fragen zum Thema Diagnose des Morbus Addison

Dieser Abschnitt bezieht sich auf die Diagnosestellung. Es sollen nur Hunde in dieser Studie eingeschlossen werden, bei denen die Diagnose zwischen 01.01.2016 - 31.10.2023 gestellt wurde.

B1. Wann wurde die Diagnose Morbus Addison gestellt?

Info: Bei unbekanntem Datum, tragen Sie 01.MM.JJJJ und den entsprechenden Monat und das jeweilige Jahr ein. Wenn zudem der Monat unklar ist, geben Sie 01.01.JJJJ und das entsprechende Jahr an.

B2. Welche Form des Hypoadrenokortizismus wurde diagnostiziert?

Primärer Hypoadrenokortizismus, einhergehend mit Veränderung des Natrium-Kaliumhaushaltes. ☐

Primärer Hypoadrenokortizismus ohne Veränderung des Natrium-Kaliumhaushaltes. ☐

Sekundärer Hypoadrenokortizismus (Störung der Hypophyse). ☐

Tertiärer Hypoadrenokortizismus (Störung des Hypothalamus). ☐

Hypoadrenokortizismus ohne Veränderung des Natrium-Kaliumhaushaltes, unklar ob es sich um einen primären, sekundären oder tertiären Hypoadrenokortizismus handelt. ☐

Keine Antwort ☐

B3. Welche/r diagnostische/n Test/s wurde/n durchgeführt?

ACTH-Stimmulationstest ☐

Kombination aus basalem Cortisol und endogenem ACTH ☐

Kombination aus basalem Cortisol und endogenem CRH ☐

Plasma-Aldosteronkonzentration (basal oder stimuliert) ☐

Aldosteron-to-renin (ARR) ratio ☐

Cortisol-to-adrenocorticotropic hormone (CAR) ratio ☐

Messung von Autoantikörpern (21-Hydroxylase-Autoantikörper) ☐

Keine Antwort ☐

B4. Welche klinischen Symptome zeigte der Patient bei Diagnosestellung?

Info: Die Akute Addison Krise ist gekennzeichnet mit einem akut auftretenden, verschlechterten Allgemeinbefinden, Dehydratation, Hypovolämie und Kollaps

Akute Addison Krise ☐

Appetitlosigkeit ☐

Gewichtsverlust ☐

Lethargie, Apathie ☐

Erbrechen ☐

Durchfall ☐

Polyurie, Polydipsie ☐

Tremor ☐

Megaoesophagus ☐

EKG-Veränderungen ☐

Keine Antwort ☐

Sonstiges:

B5. Welche Blutwertveränderung/en waren zu diesem Zeitpunkt erkennbar?

Fehlen eines Stressleukogramms (Lymphopenie, Eosinopenie, Leukoztyose, Monozytose) ☐

Hyperkaliämie ☐

Hyponatriämie ☐

Hypochloridämie ☐

Physiologische Elektrolytverteilung ☐

Azotämie ☐

Hyperphosphatämie ☐

Hypophosphatämie ☐

Hypercalcämie ☐

Hypocalcämie ☐

Hypoglykämie ☐

Hypalbuminämie ☐

Erhöhte Leberenzyme (ALT, AST) ☐

Regenerative/ aregenerative Anämie ☐

Keine Antwort ☐

Sonstiges:

Teil C: Fragen zur initialen Therapie des Morbus Addison

In diesem Abschnitt geht es um die initiale Behandlung und Glukokortikoid-Therapie des Hundes.

C1. Wurde der Hund hospitalisiert?

Der Patient wurde für mindestens 24 Stunden betreut. ☐

Der Patient wurde tagesstationär bis zur Stabilisierung betreut. ☐

Nein, der Patient wurde ambulant betreut und benötigte keine besondere Form der Überwachung. ☐

Keine Antwort ☐

C2. Wie lange war die Hospitalisierung (einschließlich der Patienten, die tagesstationär betreut wurden)?

24 Stunden ☐

48 Stunden ☐

72 Stunden ☐

96 Stunden ☐

120 Stunden ☐

> 120 Stunden ☐

Keine Antwort ☐

C3. Wenn entsprechende Elektrolytveränderungen vorlagen, wann normalisierten sich diese?

Nach 6 Stunden ☐

Nach 12 Stunden ☐

Nach 24 Stunden ☐

Nach 48 Stunden ☐

Nach 72 Stunden ☐

> 72 Stunden ☐

C4. Nach welchem Zeitraum zeigte der Patient ein ungestörtes Allgemeinbefinden?

Info: Ein ungestörtes Allgemeinbefinden beinhaltet ein eigenständiges Geh- und Stehvermögen, physiologische Vitalparameter und eine zuverlässige und eigenständige Futter- und Wasseraufnahme.

Nach 12 Stunden ☐

Nach 24 Stunden ☐

Nach 48 Stunden ☐

Nach 72 Stunden ☐

> 72 Stunden ☐

C5. Welches initiale Glukokortikoid-Präparat hat der Patient erhalten?

- Hydrocortison ☐
- Prednison ☐
- Prednisolon ☐
- Methylprednisolon ☐
- Dexamethason ☐
- Budesonid ☐
- Es wurde kein Glukokortikoid verabreicht. ☐
- Keine Antwort ☐
- ☐

Ein anderes nicht genanntes Glukokortikoid-Präparat:

C6. Hätten Sie ein anderes Glukokortikoid-Präparat gewählt, wenn dies verfügbar gewesen wäre?

- Ja ☐
- Nein ☐

C7. Welches Glukokortikoid wäre Ihr Medikament der Wahl gewesen?

- Hydrocortison ☐
- Prednison ☐
- Prednisolon ☐
- Methylprednisolon ☐
- Dexamethason ☐
- Budesonid ☐
- ☐

Ein anderes nicht genanntes Glukokortikoid-Präparat:

C8. Welche initiale Glukokortikoid-Dosierung wählten Sie bei dem Patienten? Bitte geben Sie zuerst die Dosierung in mg/kg pro Tag (d) oder im Falle einer Dauertropfinfusion in mg/kg pro Stunde (h) an. Definieren Sie dann, in wie viele Gaben die Dosierung aufgeteilt war (einmal täglich, zweimal täglich, dreimal täglich) und zuletzt geben Sie die Applikationsart (Dauertropfinfusion, intravenös (IV), intramuskulär (IM), subkutan (SC), peroral (PO)) an.

Beispiel: 0,2mg/kg/d — aufgeteilt in eine zweimal tägliche Gabe — perorale Applikation (PO)
Beispiel: 0,625 mg/kg/h —DTI — IV

Dosierung in mg/kg/Tag bzw. mg/kg/Stunde ☐☐☐ ☐ ☐☐☐☐☐

Aufgeteilt in wie viele Dosen ☐☐☐☐☐☐☐☐☐☐

Applikationsart (IV, SC, PO) ☐☐☐☐☐☐☐☐☐☐

Teil D: Im Falle einer Hospitalisierung oder einer tagesstationären Betreuung

Dieser Abschnitt bezieht sich lediglich auf die Patienten, die zu Beginn der Erkrankung hospitalisiert oder tagesstationär betreut wurden. Die Fragen konzentrieren sich auf die Therapieanpassung des Glukokortikoids im Verlaufe des stationären Aufenthaltes.

D1. Wurde eine Dosisreduktion des initialen Glukokortikoids vorgenommen?

Ja, einmalig ☐

Ja, mehrmalig ☐

Nein ☐

Keine Antwort ☐

D2. Wurde eine Dosiserhöhung des initialen Glukokortikoids vorgenommen?

Ja, einmalig ☐

Ja, mehrmalig ☐

Nein ☐

Keine Antwort ☐

D3. Wurde ein Präparatwechsel vorgenommen?

Ja, einmalig ☐

Ja, mehrmalig ☐

Nein ☐

Keine Antwort ☐

D4. Wurde eine Anpassung der Glukokortikoid-Therapie 24 Stunden nach der Einlieferung vorgenommen?

Ja ☐

Nein ☐

Keine Antwort ☐

D5. Welche Art der Anpassung wurde durchgeführt?

Dosiserhöhung ☐

Dosisreduktion ☐

Präparatwechsel ☐

Veränderung der zeitlichen Gabe (Beispiel: von zweimal täglich auf einmal täglich) ☐

Absetzen des Medikamentes ☐

Keine Antwort ☐

D6. Welches Medikament wählten Sie bei der Anpassung der Glukokortikoid-Therapie?

Hydrocortison ☐

Prednison ☐

Prednisolon ☐

Methylprednisolon ☐

Dexamethason ☐

Budesonid ☐

Ein anderes nicht genanntes Glukokortikoid-Präparat: ☐

D7. Welche angepasste Glukokortikoid-Dosierung wählten Sie bei dem Patienten nach 24 Stunden? Bitte geben Sie zuerst die Dosierung in mg/kg pro Tag (d) oder im Falle einer Dauertropfinfusion in mg/kg pro Stunde (h) an. Definieren Sie dann, in wie viele Gaben die Dosierung aufgeteilt war (einmal täglich, zweimal täglich, dreimal täglich) und zuletzt geben Sie die Applikationsart (Dauertropfinfusion, intravenös (IV), intramuskulär (IM), subkutan (SC), peroral (PO)) an.

Beispiel: 0,2mg/kg/d — aufgeteilt in eine zweimal tägliche Gabe — PO
Beispiel: 0,625 mg/kg/h — DTI — IV

Dosierung in mg/kg/Tag bzw. in mg/kg/Stunde

Aufgeteilt in wie viele Dosen

Applikationsart (IV, SC, PO)

D8. Warum wurde eine Modifikation der Therapie 24 Stunden nach der Einlieferung durchgeführt?

Klinische Beispiele, die auf eine Glukokortikoid-Überdosierung hindeuten: Polyurie und Polydipsie/ Polyphagie/ Hecheln/ Gewichtszunahme/ Depression/ Lustlosigkeit. Klinische Beispiele, die auf eine Glukokortikoid-Unterdosierung hindeuten: Anorexie/ Gewichtsverlust/ Erbrechen/ Durchfall/ Schwäche/ Mattigkeit/ Stressintoleranz.

Gutes Ansprechen auf die initiale Therapie (schnelle Verbesserung der Vital- und Laborparameter). ☐

Mäßiges Ansprechen auf die initiale Therapie (mäßige Verbesserung der Vital- und Laborparameter). ☐

Kein Ansprechen auf die initiale Therapie (keine Verbesserung der Vital- und Laborparameter). ☐

Anpassung aufgrund zu erwartender potenzieller Nebenwirkungen. ☐

Im Hinblick auf die Beeinträchtigung von einer oder mehreren bereits zuvor diagnostizierten Erkrankungen. ☐

Im Hinblick auf die Wechselwirkung mit anderen Medikamenten, die der Patient bekommt. ☐

Auftreten von Nebenwirkungen, die auf eine Glukokortikoid-Überdosierung schließen lassen. ☐

Auftreten von klinischen Anzeichen, die auf eine Glukokortikoid-Unterversorgung schließen lassen. ☐

Vorbereitung auf die häusliche Pflege. ☐

Keine Antwort ☐

☐ Sonstiges:

D9. Wie wirkten sich das Allgemeinbefinden und die klinischen Symptome auf die entsprechende Modifikation aus?

Verbesserung ☐

Verschlechterung ☐

Gleichbleibend ☐

D10. Bei bestehenden Elektrolytveränderungen (Hyponatriämie, Hyperkaliämie), wie wirkten sich diese auf die entsprechende Modifikation aus?

Verbesserung ☐

Verschlechterung ☐

Gleichbleibend ☐

D11. Wurde eine Anpassung der Glukokortikoid-Therapie 48 Stunden nach der Einlieferung vorgenommen?

Ja ☐

Nein ☐

D12. Welche Art der Anpassung wurde durchgeführt?

Dosiserhöhung ☐

Dosisreduktion ☐

Präparatwechsel ☐

Veränderung der zeitlichen Gabe (Beispiel: von zweimal täglich auf einmal täglich) ☐

Absetzen des Medikamentes ☐

Keine Antwort ☐

D13. Welches Medikament wählten Sie bei der Anpassung der Glukokortikoid-Therapie?

Hydrocortison ☐

Prednison ☐

Prednisolon ☐

Methylprednisolon ☐

Dexamethason ☐

Budesonid ☐

Ein anderes nicht genanntes Glukokortikoid-Präparat: ☐

D14. Welche angepasste Glukokortikoid-Dosierung wählten Sie bei dem Patienten nach 48 Stunden? Bitte geben Sie zuerst die Dosierung in mg/kg pro Tag (d) oder im Falle einer Dauertropfinfusion in mg/kg pro Stunde (h) an. Definieren Sie dann, in wie viele Gaben die Dosierung aufgeteilt war (einmal täglich, zweimal täglich, dreimal täglich) und zuletzt geben Sie die Applikationsart (Dauertropfinfusion, intravenös (IV), intramuskulär (IM), subkutan (SC), peroral (PO)) an.

Beispiel: 0,2mg/kg/d — aufgeteilt in eine zweimal tägliche Gabe — PO
Beispiel: 0,625 mg/kg/h — DTI — IV

Dosierung in mg/kg/Tag bzw. mg/kg/Stunde

Aufgeteilt in wie viele Dosen

Applikationsart (IV, SC, PO)

D15. Warum wurde eine Modifikation der Therapie 48 Stunden nach der Einlieferung durchgeführt?

Klinische Beispiele, die auf eine Glukokortikoid-Überdosierung hindeuten: Polyurie und Polydipsie/ Polyphagie/ Hecheln/ Gewichtszunahme/ Depression/ Lustlosigkeit. Klinische Beispiele, die auf eine Glukokortikoid-Unterdosierung hindeuten: Anorexie/ Gewichtsverlust/ Erbrechen/ Durchfall/ Schwäche/ Mattigkeit/ Stressintoleranz.

Gutes Ansprechen auf die initiale Therapie (schnelle Verbesserung der Vital- und Laborparameter). ☐

Mäßiges Ansprechen auf die initiale Therapie (mäßige Verbesserung der Vital- und Laborparameter). ☐

Kein Ansprechen auf die initiale Therapie (keine Verbesserung der Vital- und Laborparameter). ☐

Anpassung aufgrund zu erwartender potenzieller Nebenwirkungen. ☐

Im Hinblick auf die Beeinträchtigung von einer oder mehreren bereits zuvor diagnostizierten Erkrankungen. ☐

Im Hinblick auf die Wechselwirkung mit anderen Medikamenten, die der Patient bekommt. ☐

Auftreten von Nebenwirkungen, die auf eine Glukokortikoid-Überdosierung schließen lassen. ☐

Auftreten von klinischen Anzeichen, die auf eine Glukokortikoid-Unterversorgung schließen lassen. ☐

Vorbereitung auf die häusliche Pflege. ☐

Keine Antwort ☐

☐ Sonstiges:

D16. Wie wirkten sich das Allgemeinbefinden und die klinischen Symptome auf die entsprechende Modifikation aus?

Verbesserung ☐

Verschlechterung ☐

Gleichbleibend ☐

D17. Bei bestehenden Elektrolytveränderungen (Hyponatriämie, Hyperkaliämie), wie wirkten sich diese auf die entsprechende Modifikation aus?

Verbesserung ☐

Verschlechterung ☐

Gleichbleibend ☐

D18. Wurde eine Anpassung der Glukokortikoid-Therapie 72 Stunden nach der Einlieferung vorgenommen?

Ja ☐

Nein ☐

D19. Welche Art der Anpassung wurde nach 72h durchgeführt?

Dosiserhöhung ☐

Dosisreduktion ☐

Präparatwechsel ☐

Veränderung der zeitlichen Gabe (Beispiel: von zweimal täglich auf einmal täglich) ☐

Absetzen des Medikamentes ☐

Keine Antwort ☐

D20. Welches Medikament wählten Sie bei der Anpassung der Glukokortikoid-Therapie?

Hydrocortison ☐

Prednison ☐

Prednisolon ☐

Methylprednisolon ☐

Dexamethason ☐

Budesonid ☐

Ein anderes nicht genanntes Glukokortikoid-Präparat: ☐

D21. Welche angepasste Glukokortikoid-Dosierung wählten Sie bei dem Patienten nach 72 Stunden? Bitte geben Sie zuerst die Dosierung in mg/kg pro Tag (d) oder im Falle einer Dauertropfinfusion in mg/kg pro Stunde (h) an. Definieren Sie dann, in wie viele Gaben die Dosierung aufgeteilt war (einmal täglich, zweimal täglich, dreimal täglich) und zuletzt geben Sie die Applikationsart (Dauertropfinfusion, intravenös (IV), intramuskulär (IM), subkutan (SC), peroral (PO)) an.

Beispiel: 0,2mg/kg/d — aufgeteilt in eine zweimal tägliche Gabe — PO
Beispiel: 0,625 mg/kg/h — DTI — IV

Dosierung in mg/kg/Tag bzw. mg/kg/Stunde

Aufgeteilt in wie viele Dosen

Applikationsart (IV, SC, PO)

D22. Warum wurde eine Modifikation der Therapie 72 Stunden nach der Einlieferung durchgeführt?

Klinische Beispiele, die auf eine Glukokortikoid-Überdosierung hindeuten: Polyurie und Polydipsie/ Polyphagie/ Hecheln/ Gewichtszunahme/ Depression/ Lustlosigkeit. Klinische Beispiele, die auf eine Glukokortikoid-Unterdosierung hindeuten: Anorexie/ Gewichtsverlust/ Erbrechen/ Durchfall/ Schwäche/ Mattigkeit/ Stressintoleranz.

- ☐ Gutes Ansprechen auf die initiale Therapie (schnelle Verbesserung der Vital- und Laborparameter).
- ☐ Mäßiges Ansprechen auf die initiale Therapie (mäßige Verbesserung der Vital- und Laborparameter).
- ☐ Kein Ansprechen auf die initiale Therapie (keine Verbesserung der Vital- und Laborparameter).
- ☐ Anpassung aufgrund zu erwartender potenzieller Nebenwirkungen.
- ☐ Im Hinblick auf die Beeinträchtigung von einer oder mehreren bereits zuvor diagnostizierten Erkrankungen.
- ☐ Im Hinblick auf die Wechselwirkung mit anderen Medikamenten, die der Patient bekommt.
- ☐ Auftreten von Nebenwirkungen, die auf eine Glukokortikoid-Überdosierung schließen lassen.
- ☐ Auftreten von klinischen Anzeichen, die auf eine Glukokortikoid-Unterversorgung schließen lassen.
- ☐ Vorbereitung auf die häusliche Pflege.
- ☐ Keine Antwort
- ☐ Sonstiges:

D23. Wie wirkten sich das Allgemeinbefinden und die klinischen Symptome auf die entsprechende Modifikation aus?

- ☐ Verbesserung
- ☐ Verschlechterung
- ☐ Gleichbleibend

D24. Bei bestehenden Elektrolytveränderungen (Hyponatriämie, Hyperkaliämie), wie wirkten sich diese auf die entsprechende Modifikation aus?

- ☐ Verbesserung
- ☐ Verschlechterung
- ☐ Gleichbleibend

D25. Welches Medikament wählten Sie bei der Entlassung des Tieres?

- Hydrocortison ☐
- Prednison ☐
- Prednisolon ☐
- Methylprednisolon ☐
- Dexamethason ☐
- Budesonid ☐
- Es wurde kein Glukokortikoid verabreicht. ☐
- Keine Antwort ☐

Ein anderes nicht genanntes Glukokortikoid-Präparat: ☐

D26. Welche Glukokortikoid-Dosierung wählten Sie bei Entlassung des Patienten? Bitte geben Sie zuerst die Dosierung in mg/kg pro Tag (d) an. Definieren Sie dann, in wie viele Gaben die Dosierung aufgeteilt war (einmal täglich, zweimal täglich, dreimal täglich).

Beispiel: 0,2mg/kg/d — aufgeteilt in eine zweimal tägliche Gabe

Dosierung in mg/kg/Tag

Aufgeteilt in wie viele Dosen

Teil E: Fragen zur initialen Mineralokortikoid-Therapie

Dieser Abschnitt befasst sich mit der anfänglichen Mineralokortikoid-Therapie des Patienten.

E1. Wann erhielt der Patient die erste Mineralokortikoid-Supplementation?

Gleichzeitig mit Beginn der Glukokortikoid-Supplementation. ☐

Nach Bestätigung des ACTH-Stimmulationstests. ☐

Bei der Entlassung des Patienten in die häusliche Pflege. ☐

Nach 10-14 Tagen ☐

Der Patient erhält keine Mineralokortikoid-Therapie. ☐

Keine Antwort ☐

Anderer Zeitpunkt: ☐

E2. Welches initiale Mineralokortikoid-Medikament erhielt der Patient?

Info: Bei Fludrocortison handelt es sich beispielsweise um die Astonin® H 0,1 mg Tabletten (Merck Serono GmbH), während es sich bei Desoxycorticosteron pivalate (DOCP) um Zycortal® 25mg/ml Depot-Injektionssuspension (Dechra) handelt.

Fludrocortison ☐

Desoxycorticosteron pivalate (DOCP) ☐

Zunächst Fludrocortison und bei Diagnosebestätigung Umstellung auf DOCP. ☐

Keine Antwort ☐

E3. Welche initiale Fludrocortison-Dosierung wählten Sie bei dem Patienten? Bitte geben Sie zuerst die Dosierung in mg/kg pro Tag (d) an. Definieren Sie dann, in wie viele Gaben die Dosierung aufgeteilt war (einmal täglich, zweimal täglich, dreimal täglich).

Beispiel: 0,02mg/kg/d — aufgeteilt in eine zweimal tägliche Gabe

Dosierung in mg/kg/Tag

Aufgeteilt in wie viele Dosen

E4. Welche initiale DOCP-Dosierung wählten Sie bei dem Patienten? Bitte geben Sie die Dosierung in mg/kg an. Tragen Sie dann ein, nach wie vielen Tagen Sie die zweite Injektion gaben.

Beispiel: 1,5m/kg — 28 Tage

Dosierung in mg/kg

Zweite Injektion wurde wann verabreicht?

Teil F: Fragen zur Anpassung der Glukokortikoid-Therapie im Verlauf der Erkrankung

Dieser Fragenabschnitt konzentriert sich auf die Glukokortikoid-Therapiemodifikation im weiteren Krankheitsverlauf. Uns interessiert die Therapieanpassung 4 Wochen, 3 Monate, 6 Monate, 1 Jahr und 2 Jahre nach der Diagnosestellung.

F1. Wurde die Glukokortikoid-Dosierung im Verlauf der Erkrankung reduziert?

Ja, einmalig ☐

Ja, mehrmalig ☐

Nein ☐

Der Patient wird von einem/r anderen Tierarzt/in weiterbetreut. ☐

F2. Wurde die Glukokortikoid-Dosierung im Verlauf der Erkrankung längerfristig erhöht?

Ja, einmalig ☐

Ja, mehrmalig ☐

Nein ☐

Der Patient wird von einem/r anderen Tierarzt/in weiterbetreut. ☐

F3. Wurde ein Präparatwechsel im Verlauf der Therapie vorgenommen?

Beispiel: Wechsel von Prednisolon auf Hydrocortison

Ja, einmalig ☐

Ja, mehrmalig ☐

Nein ☐

Der Patient wird von einem/r anderen Tierarzt/in weiterbetreut ☐

F4. Wurde <u>4 Wochen</u> nach der Diagnose eine Anpassung der Glukokortikoid-Therapie vorgenommen?

Ja ☐

Nein ☐

Der Patient wird von einem/r anderen Tierarzt/in weiterbehandelt ☐

Der Patient ist zuvor verstorben. ☐

F5. Welche Art der Anpassung wurde nach <u>4 Wochen</u> durchgeführt?

Dosiserhöhung ☐

Dosisreduktion ☐

Präparatwechsel ☐

Veränderung der zeitlichen Gabe (Beispiel: von zweimal täglich auf einmal täglich) ☐

Absetzen des Medikamentes ☐

Keine Antwort ☐

F6. Welches Medikament wählten Sie nach <u>4 Wochen</u> bei der Anpassung der Glukokortikoid-Therapie?

Hydrocortison ☐

Prednison ☐

Prednisolon ☐

Methylprednisolon ☐

Dexamethason ☐

Budesonid ☐

☐ Ein anderes nicht genanntes Glukokortikoid-Präparat:

F7. Welche Glukokortikoid-Dosierung wählten Sie bei der Anpassung des Patienten <u>nach 4 Wochen</u>? Bitte geben Sie zuerst die Dosierung in mg/kg pro Tag (d) an. Definieren Sie dann, in wie viele Gaben die Dosierung aufgeteilt war (einmal täglich, zweimal täglich, dreimal täglich).

Beispiel: 0,1 mg/kg/d — einmal tägliche Gabe

Dosierung in mg/kg/Tag										
Aufgeteilt in wie viele Gaben										

F8. Warum wurde eine Modifikation der Therapie <u>4 Wochen</u> nach der Diagnosestellung durchgeführt?

Klinische Anzeichen, welche auf eine Glukokortikoid-Überdosierung hindeuten: Polyurie und Polydipsie/ Polyphagie/ Hecheln/ Gewichtszunahme/ Depression/ Lustlosigkeit/ Haarverlust/ trockene, feste Haut/ Muskelschwund/ Zunahme Bauchumfang.
Klinische Anzeichen, welche auf eine Glukokortikoid-Unterdosierung hindeuten: Anorexie/ Gewichtsverlust/ Erbrechen/ Durchfall/ abdominale Schmerzen/ Schwäche/ Mattigkeit/ Stressintoleranz.

Findung der optimalen Dosis (geringste effektive Dosis). ☐

Anpassung aufgrund zu erwartender potenzieller Nebenwirkungen. ☐

Mäßiges Ansprechen auf die Therapie (mäßige Verbesserung der Vital- und Laborparameter). ☐

Kein Ansprechen auf die Therapie (keine Verbesserung der Vital- und Laborparameter). ☐

Im Hinblick auf die Beeinträchtigung von einer oder mehreren bereits zuvor diagnostizierten Erkrankungen. ☐

☐

Im Hinblick auf die Wechselwirkung mit anderen Medikamenten, die der Patient bekommt.

Auftreten von Nebenwirkungen, die auf eine Glukokortikoid-Überdosierung schließen lassen.

Auftreten von klinischen Anzeichen, die auf eine Glukokortikoid-Unterversorgung schließen lassen.

Keine Antwort

Sonstiges:

F9. Wie wirkten sich das Allgemeinbefinden und die klinischen Symptome auf die entsprechende Modifikation aus?

Verbesserung

Verschlechterung

Gleichbleibend

F10. Wurde 3 Monate nach der Diagnose eine Anpassung der Glukokortikoid-Therapie vorgenommen?

Ja

Nein

Der Patient wird von einem/r anderen Tierarzt/in weiterbetreut.

Der Patient ist zuvor verstorben.

F11. Welche Art der Anpassung wurde nach 3 Monaten durchgeführt?

Dosiserhöhung

Dosisreduktion

Präparatwechsel

Veränderung der zeitlichen Gabe (Beispiel: von zweimal täglich auf einmal täglich)

Absetzen des Medikamentes

Keine Antwort

F12. Welches Medikament wählten Sie nach 3 Monaten bei der Anpassung der Glukokortikoid-Therapie?

Hydrocortison

Prednison

Prednisolon

Methylprednisolon ☐

Dexamethason ☐

Budesonid ☐

Ein anderes nicht genanntes Glukokortikoid-Präparat: ☐

F13. Welche Glukokortikoid-Dosierung wählten Sie bei der Anpassung des Patienten nach 3 Monaten? Bitte geben Sie zuerst die Dosierung in mg/kg pro Tag (d) an. Definieren Sie dann, in wie viele Gaben die Dosierung aufgeteilt war (einmal täglich, zweimal täglich, dreimal täglich).

Beispiel: 0,1 mg/kg/d — einmal tägliche Gabe

Dosierung in mg/kg/Tag

Aufgeteilt in wie viele Gaben

F14. Warum wurde eine Modifikation der Therapie 3 Monate nach der Diagnosestellung durchgeführt?

Klinische Anzeichen, welche auf eine Glukokortikoid-Überdosierung hindeuten: Polyurie und Polydipsie/ Polyphagie/ Hecheln/ Gewichtszunahme/ Depression/ Lustlosigkeit/ Haarverlust/ trockene, feste Haut/ Muskelschwund/ Zunahme Bauchumfang. Klinische Anzeichen, welche auf eine Glukokortikoid-Unterdosierung hindeuten: Anorexie/ Gewichtsverlust/ Erbrechen/ Durchfall/ abdominale Schmerzen/ Schwäche/ Mattigkeit/ Stressintoleranz.

Findung der optimalen Dosis (geringste effektive Dosis). ☐

Anpassung aufgrund zu erwartender potenzieller Nebenwirkungen. ☐

Mäßiges Ansprechen auf die Therapie (mäßige Verbesserung der Vital- und Laborparameter). ☐

Kein Ansprechen auf die Therapie (keine Verbesserung der Vital- und Laborparameter). ☐

Im Hinblick auf die Beeinträchtigung von einer oder mehreren bereits zuvor diagnostizierten Erkrankungen. ☐

Im Hinblick auf die Wechselwirkung mit anderen Medikamenten, die der Patient bekommt. ☐

Auftreten von Nebenwirkungen, die auf eine Glukokortikoid-Überdosierung schließen lassen. ☐

Auftreten von klinischen Anzeichen, die auf eine Glukokortikoid-Unterversorgung schließen lassen. ☐

Keine Antwort

Sonstiges: ☐

F15. Wie wirkten sich das Allgemeinbefinden und die klinischen Symptome auf die entsprechende Modifikation aus?

Verbesserung ☐

Verschlechterung ☐

Gleichbleibend ☐

F16. Wurde <u>6 Monate</u> nach der Diagnose eine Anpassung der Glukokortikoid-Therapie vorgenommen?

Ja ☐

Nein ☐

Der Patient wird von einem/r anderen Tierarzt/in weiterbetreut. ☐

Der Patient ist zuvor verstorben. ☐

F17. Welche Art der Anpassung wurde nach <u>6 Monaten</u> durchgeführt?

Dosiserhöhung ☐

Dosisreduktion ☐

Präparatwechsel ☐

Veränderung der zeitlichen Gabe (Beispiel: von zweimal täglich auf einmal täglich) ☐

Absetzen des Medikamentes ☐

Keine Antwort ☐

F18. Welches Medikament wählten Sie nach <u>6 Monaten</u> bei der Anpassung der Glukokortikoid-Therapie?

Hydrocortison ☐

Prednison ☐

Prednisolon ☐

Methylprednisolon ☐

Dexamethason ☐

Budesonid ☐

☐ Ein anderes nicht genanntes Glukokortikoid-Präparat:

F19. Welche Glukokortikoid-Dosierung wählten Sie bei der Anpassung des Patienten nach <u>6 Monaten</u>? Bitte geben Sie zuerst die Dosierung in mg/kg pro Tag (d) an. Definieren Sie dann, in wie viele Gaben die Dosierung aufgeteilt war (einmal täglich, zweimal täglich, dreimal täglich).

Beispiel: 0,1 mg/kg/d — einmal tägliche Gabe

Dosierung in mg/kg/Tag ☐☐☐☐☐☐☐☐☐☐

Aufgeteilt in wie viele Gaben ☐☐☐☐☐☐☐☐☐☐

F20. Warum wurde eine Modifikation der Therapie <u>6 Monate</u> nach der Diagnosestellung durchgeführt?

Klinische Anzeichen, welche auf eine Glukokortikoid-Überdosierung hindeuten: Polyurie und Polydipsie/ Polyphagie/ Hecheln/ Gewichtszunahme/ Depression/ Lustlosigkeit/ Haarverlust/ trockene, feste Haut/ Muskelschwund/ Zunahme Bauchumfang.
Klinische Anzeichen, welche auf eine Glukokortikoid-Unterdosierung hindeuten: Anorexie/ Gewichtsverlust/ Erbrechen/ Durchfall/ abdominale Schmerzen/ Schwäche/ Mattigkeit/ Stressintoleranz.

Findung der optimalen Dosis (geringste effektive Dosis). ☐

Anpassung aufgrund zu erwartender potenzieller Nebenwirkungen. ☐

Mäßiges Ansprechen auf die Therapie (mäßige Verbesserung der Vital- und Laborparameter). ☐

Kein Ansprechen auf die Therapie (keine Verbesserung der Vital- und Laborparameter). ☐

Im Hinblick auf die Beeinträchtigung von einer oder mehreren bereits zuvor diagnostizierten Erkrankungen. ☐

Im Hinblick auf die Wechselwirkung mit anderen Medikamenten, die der Patient bekommt. ☐

Auftreten von Nebenwirkungen, die auf eine Glukokortikoid-Überdosierung schließen lassen. ☐

Auftreten von klinischen Anzeichen, die auf eine Glukokortikoid-Unterversorgung schließen lassen. ☐

Keine Antwort

Sonstiges: ☐

F21. Wie wirkten sich das Allgemeinbefinden und die klinischen Symptome auf die entsprechende Modifikation aus?

Verbesserung ☐

Verschlechterung ☐

Gleichbleibend ☐

F22. Wurde <u>1 Jahr</u> nach der Diagnose eine Anpassung der Glukokortikoid-Therapie vorgenommen?

☐

Ja

Nein ☐

Der Patient wird von einem/r anderen Tierarzt/in weiterbetreut. ☐

Der Patient ist zuvor verstorben. ☐

F23. Welche Art der Anpassung wurde nach 1 Jahr durchgeführt?

Dosiserhöhung ☐

Dosisreduktion ☐

Präparatwechsel ☐

Veränderung der zeitlichen Gabe (Beispiel: von zweimal täglich auf einmal täglich) ☐

Absetzen des Medikamentes ☐

Keine Antwort ☐

F24. Welches Medikament wählten Sie nach 1 Jahr bei der Anpassung der Glukokortikoid-Therapie?

Hydrocortison ☐

Prednison ☐

Prednisolon ☐

Methylprednisolon ☐

Dexamethason ☐

Budesonid ☐

Ein anderes nicht genanntes Glukokortikoid-Präparat: ☐

F25. Welche Glukokortikoid-Dosierung wählten Sie bei der Anpassung des Patienten nach 1 Jahr? Bitte geben Sie zuerst die Dosierung in mg/kg pro Tag (d) an. Definieren Sie dann, in wie viele Gaben die Dosierung aufgeteilt war (einmal täglich, zweimal täglich, dreimal täglich).

Beispiel: 0,1 mg/kg/d — einmal tägliche Gabe

Dosierung in mg/kg/Tag

Aufgeteilt in wie viele Gaben

F26. Warum wurde eine Modifikation der Therapie 1 Jahr nach der Diagnosestellung durchgeführt?

Klinische Anzeichen, welche auf eine Glukokortikoid-Überdosierung hindeuten: Polyurie und Polydipsie/ Polyphagie/ Hecheln/ Gewichtszunahme/ Depression/ Lustlosigkeit/ Haarverlust/ trockene, feste Haut/ Muskelschwund/ Zunahme Bauchumfang.
Klinische Anzeichen, welche auf eine Glukokortikoid-Unterdosierung hindeuten: Anorexie/ Gewichtsverlust/ Erbrechen/ Durchfall/ abdominale Schmerzen/ Schwäche/ Mattigkeit/ Stressintoleranz.

Findung der optimalen Dosis (geringste effektive Dosis). ☐

Anpassung aufgrund zu erwartender potenzieller Nebenwirkungen. ☐

Mäßiges Ansprechen auf die Therapie (mäßige Verbesserung der Vital- und Laborparameter). ☐

Kein Ansprechen auf die Therapie (keine Verbesserung der Vital- und Laborparameter). ☐

Im Hinblick auf die Beeinträchtigung von einer oder mehreren bereits zuvor diagnostizierten. Erkrankungen ☐

Im Hinblick auf die Wechselwirkung mit anderen Medikamenten, die der Patient bekommt. ☐

Auftreten von Nebenwirkungen, die auf eine Glukokortikoid-Überdosierung schließen lassen. ☐

Auftreten von klinischen Anzeichen, die auf eine Glukokortikoid-Unterversorgung schließen lassen. ☐

Keine Antwort ☐

Sonstiges:

F27. Wie wirkten sich das Allgemeinbefinden und die klinischen Symptome auf die entsprechende Modifikation aus?

Verbesserung ☐

Verschlechterung ☐

Gleichbleibend ☐

F28. Wurde 2 Jahre post Diagnose eine Anpassung der Glukokortikoid-Therapie vorgenommen?

Ja ☐

Nein ☐

Der Patient wird von einem/r anderen Tierarzt/in weiterbetreut. ☐

Der Patient ist zuvor verstorben. ☐

F29. Welche Art der Anpassung wurde nach 2 Jahren durchgeführt?

Dosiserhöhung ☐

Dosisreduktion ☐

Präparatwechsel ☐

Veränderung der zeitlichen Gabe (Beispiel: von zweimal täglich auf einmal täglich) ☐

Absetzen des Medikamentes ☐

Keine Antwort ☐

F30. Welches Medikament wählten Sie nach 2 Jahren bei der Anpassung der Glukokortikoid-Therapie?

Hydrocortison ☐

Prednison ☐

Prednisolon ☐

Methylprednisolon ☐

Dexamethason ☐

Budesonid ☐

☐ Ein anderes nicht genanntes Glukokortikoid-Präparat:

F31. Welche Glukokortikoid-Dosierung wählten Sie bei der Anpassung des Patienten nach 2 Jahren? Bitte geben Sie zuerst die Dosierung in mg/kg pro Tag (d) an. Definieren Sie dann, in wie viele Gaben die Dosierung aufgeteilt war (einmal täglich, zweimal täglich, dreimal täglich).

Beispiel: 0,1 mg/kg/d — einmal tägliche Gabe

Dosierung in mg/kg/Tag

Aufgeteilt in wie viele Gaben

F32. Warum wurde eine Modifikation der Therapie 2 Jahre nach der Diagnosestellung durchgeführt?

Klinische Anzeichen, welche auf eine Glukokortikoid-Überdosierung hindeuten: Polyurie und Polydipsie/ Polyphagie/ Hecheln/ Gewichtszunahme/ Depression/ Lustlosigkeit/ Haarverlust/ trockene, feste Haut/ Muskelschwund/ Zunahme Bauchumfang.
Klinische Anzeichen, welche auf eine Glukokortikoid-Unterdosierung hindeuten: Anorexie/ Gewichtsverlust/ Erbrechen/ Durchfall/ abdominale Schmerzen/ Schwäche/ Mattigkeit/ Stressintoleranz.

Findung der optimalen Dosis (geringste effektive Dosis). ☐

Anpassung aufgrund zu erwartender potenzieller Nebenwirkungen. ☐

Mäßiges Ansprechen auf die Therapie (mäßige Verbesserung der Vital- und Laborparameter). ☐

Kein Ansprechen auf die Therapie (keine Verbesserung der Vital- und Laborparameter). ☐

Im Hinblick auf die Beeinträchtigung von einer oder mehreren bereits zuvor diagnostizierten Erkrankungen. ☐

Im Hinblick auf die Wechselwirkung mit anderen Medikamenten, die der Patient bekommt. ☐

Auftreten von Nebenwirkungen, die auf eine Glukokortikoid-Überdosierung schließen lassen. ☐

Auftreten von klinischen Anzeichen, die auf eine Glukokortikoid-Unterversorgung schließen lassen. ☐

Keine Antwort ☐

Sonstiges:

F33. Wie wirkten sich das Allgemeinbefinden und die klinischen Symptome auf die entsprechende Modifikation aus?

Verbesserung ☐

Verschlechterung ☐

Gleichbleibend ☐

Teil G: Fragen zur aktuellen bzw. zuletzt bekannten Glukokortikoid-Therapie

In diesem Teil geht es um die aktuelle Glukokortikoid-Dosierung Ihres Patienten. Falls der Hund bereits verstorben sein sollte, geben Sie die Ihnen zuletzt bekannte Glukokortikoid-Therapie an.

G1. Wie lautet das aktuelle bzw. zuletzt bekannte Glukokortikoid- Medikament, welches der Patient bekommt bzw. bekam?

Info: Falls der Patient bereits verstorben sein sollte, wählen Sie bitte das zuletzt gegebene Medikament aus

Hydrocortison ☐

Prednison ☐

Prednisolon ☐

Methylprednisolon ☐

Dexamethason ☐

Budesonid ☐

Der Patient wird von einem/r anderen Tierarzt/in weiterbetreut. ☐

Das Glukokortikoid-Medikament wurde abgesetzt. ☐

Ein anderes nicht genanntes Glukokortikoid-Präparat: ☐

G2. Welche aktuelle bzw. zuletzt bekannte Dosierung erhält bzw. erhielt der Patient? Bitte geben Sie zuerst die Dosierung in mg/kg pro Tag (d) an. Definieren Sie dann, in wie viele Gaben die Dosierung aufgeteilt ist bzw. war (einmal täglich, zweimal täglich, dreimal täglich).

Info: Falls der Patient bereits verstorben sein sollte, wählen Sie bitte die zuletzt gegebene Dosierung aus
Beispiel: 0,1 mg/kg/d — einmal tägliche Gabe

Dosierung in mg/kg/Tag

Aufgeteilt in wie viele Gaben

G3. Ist bzw. war der Morbus Addison hinsichtlich der aktuellen bzw. zuletzt bekannten Glukokortikoid-Dosierung nach Ihrer Ansicht optimal eingestellt?

Info: Eine optimale Einstellung wird bemessen anhand des Allgemeinbefindens, der Vitalparameter und dem Vorhandensein von Nebenwirkungen die auf eine Unter- oder Überdosierung schließen lassen.

Der Hund ist bzw. war optimal eingestellt. ☐

Der Hund ist bzw. war gut eingestellt. ☐

Der Hund ist bzw. war mäßig eingestellt. ☐

Der Hund ist bzw. war schlecht eingestellt. ☐

Der Hund ist bzw. war sehr schlecht eingestellt. ☐

Keine Aussage möglich, da der Hund noch andere Erkrankungen aufweist, die das klinische Bild verschlechtern ☐

G4. Verwenden bzw. verwendeten Sie zur Optimierung der Glukokortikoid-Substitutionstherapie die Testung des basalen ACTHs?

Ja, der Test erwies sich als hilfreich. ☐

Ja, allerdings war der Test nicht nützlich. ☐

Nein ☐

Teil H: Fragen zur Glukokortikoid-Dosierung Ihres Patienten in stressigen Situationen

In diesem Abschnitt möchten wir von Ihnen wissen, wie Sie die Glukokortikoid-Therapie Ihres Patienten in Situationen mit Stress handhaben.

H1. Empfehlen Sie dem Patientenbesitzer kurzzeitig die Glukokortikoid-Dosierung in Situationen mit Stress zu erhöhen?

Ja, bei jeglicher Art von Stresssituationen (Urlaub, Tierarztbesuch, Hitze). ☐

Ja, nur bei einer außergewöhnlichen Stresssituation (Silvester, Feuerwerk). ☐

Nein, ich rate nicht zu einer Erhöhung der Glukokortikoid-Dosierung in einer stressigen Situation. ☐

H2. Wenn Sie zu einer Erhöhung der Glukokortikoid-Dosierung in Stresssituationen raten, wie hoch fällt diese durchschnittlich aus?

10% ☐

25% ☐

50% ☐

75% ☐

100% ☐

Andere Angabe: ☐

H3. Welchen durchschnittlichen Zeitraum empfehlen Sie zur Erhöhung der Glukokortikoid-Dosierung im Falle einer Stresssituation?

< 2 Tage ☐

2 Tage ☐

3 Tage ☐

4 Tage ☐

7 Tage ☐

Anderer Zeitraum: ☐

H4. In welchem Zeitraum reduzieren Sie die erhöhte Glukokortikoid-Dosierung durchschnittlich nach einer stressigen Situation?

< 2 Tage ☐

2 Tage ☐

3 Tage ☐

4 Tage ☐

7 Tage ☐

☐ Anderer Zeitraum:

Teil I: Fragen zur Anpassung der Mineralokortikoid-Therapie im Verlauf der Erkrankung

In diesem Teil geht es um die Mineralokortikoid-Therapieanpassung im weiteren Krankheitsverlauf.

I1. Wurde die Mineralokortikoid-Supplementation im Laufe der Erkrankung angepasst?

Ja, einmalig ☐

Ja, mehrmalig ☐

Nein ☐

Das Medikament wurde abgesetzt. ☐

Der Patient erhält keine Mineralokortikoid-Supplementation. ☐

Der Patient wird von einem/r anderen Tierarzt/in weiterbetreut. ☐

I2. Wurde die Mineralokortikoid-Supplementation im Verlaufe der Erkrankung reduziert?

Ja, einmalig ☐

Ja, mehrmalig ☐

Nein ☐

I3. Wurde die Mineralokortikoid-Supplementation im Verlaufe der Erkrankung erhöht?

Ja, einmalig ☐

Ja, mehrmalig ☐

Nein ☐

I4. Wurde das Präparat im Verlaufe der Erkrankung gewechselt?

Info: Bei Fludrocortison handelt es sich beispielsweise um die Astonin® H 0,1 mg Tabletten (Merck Serono GmbH), während es sich bei Desoxycorticosteron pivalate (DOCP) um Zycortal® 25mg/ml Depot-Injektionssuspension (Dechra) handelt

- ☐ Ja, von DOCP auf Fludrocortison.
- ☐ Ja, von Fludrocortison auf DOCP.
- ☐ Nein, der Patient erhält weiterhin Fludrocortison.
- ☐ Nein, der Patient erhält weiterhin DOCP.

I5. Warum wurde ein Präparatwechsel im Verlaufe der Erkrankung durchgeführt?

- ☐ Anhaltende Elektrolytabweichungen, die für eine Mineralokortikoid-Unterdosierung sprechen (Hyperkaliämie, Hyponatriämie).
- ☐ Anhaltende Elektrolytabweichungen, die für eine Mineralokortikoid-Überdosierung sprechen (Hypokaliämie, Hypernatriämie).
- ☐ Klinische Abnormitäten
- ☐ Medikamentenunverträglichkeit
- ☐ Keine Antwort
- ☐ Sonstiges:

I6. Wie lautet die aktuelle bzw. zuletzt bekannte Fludrocortison-Dosierung bei dem Patienten? Bitte geben Sie zuerst die Dosierung in mg/kg pro Tag (d) an. Definieren Sie dann, in wie viele Gaben die Dosierung aufgeteilt ist bzw. war (einmal täglich, zweimal täglich, dreimal täglich).

Info: Bei Fludrocortison handelt es sich beispielsweise um die Astonin® H 0,1 mg Tabletten (Merck Serono GmbH)
Beispiel: 0,02mg/kg/d — aufgeteilt in eine zweimal tägliche Gabe

Dosierung in mg/kg/Tag

Aufgeteilt in wie viele Gaben

I7. Wie lautet die aktuelle bzw. zuletzt bekannte DOCP-Dosierung bei dem Patienten? Bitte geben Sie die Dosierung in mg/kg an. Tragen Sie dann ein, nach wie vielen Tagen Sie die nächste Injektion geben bzw. gaben.

Info: Bei Desoxycorticosteron pivalate (DOCP) handelt es sich um Zycortal® 25mg/ml Depot-Injektionssuspension (Dechra)
Beispiel: 1,5m/kg — 28 Tage

Dosierung in mg/kg

Nächste Injektion nach wie vielen Tagen?

Teil J: Fragen zu anderen Erkrankungen und Komorbiditäten des Patienten

In dem letzten Fragenabschnitt geht es um weitere Erkrankungen, an die Ihr Patient möglicherweise leidet bzw. litt. Wir möchten uns dabei lediglich auf endokrinologische, immunvermittelte und/ oder entzündlichen Erkrankungen fokussieren.

J1. Leidet bzw. litt der Patient an einer oder mehreren weiteren endokrinologischen, immunvermittelten oder entzündlichen Erkrankung/en?

Info: Endokrinologische Erkrankung z.B. Hypothyreose, Immunvermittelte Erkrankung z.B. primär immunvermittelte Anämie, Entzündliche Erkrankung z.B. Pankreatitis

- Der Patient hat bzw. hatte eine weitere Erkrankung. ☐
- Der Patient hat bzw. hatte zwei weitere Erkrankungen. ☐
- Der Patient hat bzw. hatte drei weitere Erkrankungen. ☐
- Der Patient hat bzw. hatte > drei weitere Erkrankungen. ☐
- Nein, der Patient leidet bzw. litt an keiner weiteren Erkrankung. ☐
- Der Patient wird bzw. wurde von einem/r anderen Tierarzt/in weiterbetreut. ☐

J2. Wie lautet die erste Erkrankung, an welcher der Patient leidet bzw. litt?

- Hypothyreose ☐
- Hypoparathyreoidismus ☐
- Hyperparathyreoidismus ☐
- Diabetes mellitus ☐
- Diabetes insipidus ☐
- Primär immunvermittelte Anämie ☐
- Primär immunvermittelte Thrombozytopenie ☐
- Azoospermie ☐
- Glomerulopathie ☐
- Pankreatitis ☐
- Futtermittel-Responsive-Enteropathie ☐
- Immunsuppressiva-Responsive-Enteropathie ☐
- Andere Erkrankung: ☐

J3. Wann wurde die <u>erste Erkrankung</u> diagnostiziert?

Vor der Diagnosestellung des Hypoadrenokortizismus ☐

Zum gleichen Zeitpunktion mit der Diagnosestellung Hypoadrenokortizismus ☐

Nach der Diagnosestellung des Hypoadrenokortizismus ☐

J4. Wurde die <u>erste Erkrankung</u> erfolgreich behandelt oder besteht bzw. bestand weiterhin?

Die Erkrankung wurde erfolgreich behandelt und der Patient bekommt bzw. bekam keine Medikamente mehr. ☐

Die Erkrankung ist bzw. war unter Therapie und der Patient ist bzw. war. damit gut eingestellt. ☐

Die Erkrankung ist bzw. war unter Therapie und der Patient hat bzw. hatte geringgradige Beschwerden. ☐

Die Erkrankung ist bzw. war unter Therapie und der Patient hat bzw. hatte mittelgradige Beschwerden. ☐

Die Erkrankung ist bzw. war unter Therapie und der Patient hat bzw. hatte hochgradige Beschwerden. ☐

Der Patient ist an der Erkrankung verstorben oder wurde diesbezüglich euthanasiert. ☐

J5. Welche/s Medikament/e bekommt bzw. bekam der Patient zur Therapie der <u>ersten Erkrankung</u>?

Info: Bitte geben Sie das oder das Medikament/e manuell ein

J6. Wie lautet die <u>zweite Erkrankung,</u> an welcher der Patient leidet bzw. litt?

Hypothyreose ☐

Hypoparathyreoidismus ☐

Hyperparathyreoidismus ☐

Diabetes mellitus ☐

Diabetes insipidus ☐

Primär immunvermittelte Anämie ☐

Primär immunvermittelte Thrombozytopenie ☐

Azoospermie ☐

Glomerulopathie ☐

Pankreatitis ☐

☐

Futtermittel-Responsive-Enteropathie ☐

Immunsuppressiva-Responsive-Enteropathie ☐

Andere Erkrankung:

J7. Wann wurde die <u>zweite Erkrankung</u> an, welche der Patient leidet bzw. litt diagnostiziert?

Vor der Diagnosestellung des Hypoadrenokortizismus ☐

Zum gleichen Zeitpunktion mit der Diagnosestellung Hypoadrenokortizismus ☐

Nach der Diagnosestellung des Hypoadrenokortizismus ☐

J8. Wurde die <u>zweite Erkrankung</u> erfolgreich behandelt oder besteht sie weiterhin?

Die Erkrankung wurde erfolgreich behandelt und der Patient bekommt bzw. bekam keine Medikamente mehr. ☐

Die Erkrankung ist bzw. war unter Therapie und der Patient ist bzw. war. damit gut eingestellt. ☐

Die Erkrankung ist bzw. war unter Therapie und der Patient hat bzw. hatte geringgradige Beschwerden. ☐

Die Erkrankung ist bzw. war unter Therapie und der Patient hat bzw. hatte mittelgradige Beschwerden. ☐

Die Erkrankung ist bzw. war unter Therapie und der Patient hat bzw. hatte hochgradige Beschwerden. ☐

Der Patient ist an der Erkrankung verstorben oder wurde diesbezüglich euthanasiert. ☐

J9. Welche/s Medikament/e bekommt bzw. bekam der Patient zur Therapie der <u>zweiten Erkrankung</u>?

Info: Bitte geben Sie das oder das Medikament/e manuell ein

J10. Wie lautet die <u>dritte Erkrankung,</u> an welcher der Patient leidet bzw. litt?

Hypothyreose ☐

Hypoparathyreoidismus ☐

Hyperparathyreoidismus ☐

☐

Diabetes mellitus

Diabetes insipidus ☐

Primär immunvermittelte Anämie ☐

Primär immunvermittelte Thrombozytopenie ☐

Azoospermie ☐

Glomerulopathie ☐

Pankreatitis ☐

Futtermittel-Responsive-Enteropathie ☐

Immunsuppressiva-Responsive-Enteropathie ☐

Andere Erkrankung: ☐

J11. Wann wurde die <u>dritte Erkrankung</u> an, welche der Patient leidet bzw. litt diagnostiziert?

Vor der Diagnosestellung des Hypoadrenokortizismus ☐

Zum gleichen Zeitpunktion mit der Diagnosestellung Hypoadrenokortizismus ☐

Nach der Diagnosestellung des Hypoadrenokortizismus ☐

J12. Wurde die <u>dritte Erkrankung</u> erfolgreich behandelt oder besteht sie weiterhin?

Die Erkrankung wurde erfolgreich behandelt und der Patient bekommt bzw. bekam keine Medikamente mehr. ☐

Die Erkrankung ist bzw. war unter Therapie und der Patient ist bzw. war. damit gut eingestellt. ☐

Die Erkrankung ist bzw. war unter Therapie und der Patient hat bzw. hatte geringgradige Beschwerden. ☐

Die Erkrankung ist bzw. war unter Therapie und der Patient hat bzw. hatte mittelgradige Beschwerden. ☐

Die Erkrankung ist bzw. war unter Therapie und der Patient hat bzw. hatte hochgradige Beschwerden. ☐

Der Patient ist an der Erkrankung verstorben oder wurde diesbezüglich euthanasiert. ☐

J13. Welche/s Medikament/e bekommt bzw. bekam der Patient zur Therapie der <u>dritten Erkrankung</u>?

Info: Bitte geben Sie das oder das Medikament/e manuell ein

J14. Wie lautet die <u>vierte Erkrankung,</u> an welcher der Patient leidet bzw. litt?

Info: Sie haben angegeben, dass der Patient an mehr als drei zusätzliche endokrinologische, immunvermittelte und/oder entzündliche Erkrankungen leidet. Bitte beschreiben Sie die vier Erkrankungen, mit dem höchsten Stellenwert.

- Hypothyreose ☐
- Hypoparathyreoidismus ☐
- Hyperparathyreoidismus ☐
- Diabetes mellitus ☐
- Diabetes insipidus ☐
- Primär immunvermittelte Anämie ☐
- Primär immunvermittelte Thrombozytopenie ☐
- Azoospermie ☐
- Glomerulopathie ☐
- Pankreatitis ☐
- Futtermittel-Responsive-Enteropathie ☐
- Immunsuppressiva-Responsive-Enteropathie ☐
- Andere Erkrankung: ☐

J15. Wann wurde die <u>vierte Erkrankung</u> an, welche der Patient leidet bzw. litt diagnostiziert?

- Vor der Diagnosestellung des Hypoadrenokortizismus ☐
- Zum gleichen Zeitpunktion mit der Diagnosestellung Hypoadrenokortizismus ☐
- Nach der Diagnosestellung des Hypoadrenokortizismus ☐

J16. Wurde die vierte Erkrankung erfolgreich behandelt oder besteht sie weiterhin?

Die Erkrankung wurde erfolgreich behandelt und der Patient bekommt bzw. bekam keine Medikamente mehr. ☐

Die Erkrankung ist bzw. war unter Therapie und der Patient ist bzw. war. damit gut eingestellt. ☐

Die Erkrankung ist bzw. war unter Therapie und der Patient hat bzw. hatte geringgradige Beschwerden. ☐

Die Erkrankung ist bzw. war unter Therapie und der Patient hat bzw. hatte mittelgradige Beschwerden. ☐

Die Erkrankung ist bzw. war unter Therapie und der Patient hat bzw. hatte hochgradige Beschwerden. ☐

Der Patient ist an der Erkrankung verstorben oder wurde diesbezüglich euthanasiert. ☐

J17. Welche/s Medikament/e bekommt bzw. bekam der Patient zur Therapie der vierten Erkrankung?

Info: Bitte geben Sie das oder das Medikament/e manuell ein

Vielen Dank für die Teilnahme an der Umfrage!
